# *Bacillaceae* serine proteases and *Streptomyces* epsilon-poly-l-lysine synergistically inactivate *Caliciviridae* by inhibiting RNA genome release

**DOI:** 10.1038/s41598-024-65963-9

**Published:** 2024-07-02

**Authors:** Soh Yamamoto, Noriko Ogasawara, Yuka Sudo-Yokoyama, Sachiko Sato, Nozomu Takata, Nana Yokota, Tomomi Nakano, Kyoko Hayashi, Akira Takasawa, Mayumi Endo, Masako Hinatsu, Keitaro Yoshida, Toyotaka Sato, Satoshi Takahashi, Kenichi Takano, Takashi Kojima, Jun Hiraki, Shin-ich Yokota

**Affiliations:** 1 Department of Microbiology, Sapporo Medical University School of Medicine, Sapporo, 060-8556 Japan; 2 Department of Otolaryngology-Head and Neck Surgery, Sapporo Medical University School of Medicine, Sapporo, 060-8556 Japan; 3 Center for Vascular and Developmental Biology, Feinberg Cardiovascular and Renal Research Institute, Feinberg School of Medicine, Northwestern University, Chicago, IL 60611 USA; 4 Yokohama R&D Center, JNC Corporation, Yokohama, 236-8605 Japan; 5 College of Life and Health Sciences, Chubu University, Kasugai, 487-8501 Japan; 6 Department of Pathology, Asahikawa Medical University, Asahikawa, 078-8510 Japan; 7 Laboratory of Veterinary Hygiene, Faculty of Veterinary Medicine, Hokkaido University, Sapporo, 060-0818 Japan; 8 Graduate School of Infectious Diseases, Hokkaido University, Sapporo, 060-0818 Japan; 9 One Health Research Center, Hokkaido University, Sapporo, 060-0818 Japan; 10 Department of Infection Control and Laboratory Medicine, Sapporo Medical University School of Medicine, Sapporo, 060-8556 Japan; 11 Department of Cell Science, Sapporo Medical University School of Medicine, Sapporo, 060-8556 Japan

**Keywords:** Human norovirus, *Caliciviridae*, *Bacillaceae* serine proteases, Epsilon-poly-l-lysine, Natural products, Infection, Virology, Viral transmission

## Abstract

Human norovirus (HuNoV) is an enteric infectious pathogen belonging to the *Caliciviridae* family that causes occasional epidemics. Circulating alcohol-tolerant viral particles that are readily transmitted via food-borne routes significantly contribute to the global burden of HuNoV-induced gastroenteritis. Moreover, contact with enzymes secreted by other microorganisms in the environment can impact the infectivity of viruses. Hence, understanding the circulation dynamics of *Caliciviridae* is critical to mitigating epidemics. Accordingly, in this study, we screened whether environmentally abundant secretase components, particularly proteases, affect *Caliciviridae* infectivity. Results showed that combining *Bacillaceae* serine proteases with epsilon-poly-l-lysine (EPL) produced by *Streptomyces—*a natural antimicrobial—elicited anti-*Caliciviridae* properties, including against the epidemic HuNoV GII.4_Sydney_2012 strain*. *In vitro and in vivo biochemical and virological analyses revealed that EPL has two unique synergistic viral inactivation functions. First, it maintains an optimal pH to promote viral surface conformational changes to the protease-sensitive structure. Subsequently, it inhibits viral RNA genome release via partial protease digestion at the P2 and S domains in the VP1 capsid. This study provides new insights regarding the high-dimensional environmental interactions between bacteria and *Caliciviridae*, while promoting the development of protease-based anti-viral disinfectants.

## Introduction

Natural environments contain a multitude of diverse microorganisms, the delicate interactions among which greatly impact all living things, including humans, whether latent or overt, direct or indirect^1^. Hence, an in-depth understanding of the interkingdom interactions between viruses and bacteria—representative microorganisms—is necessary to fully characterize the complex life cycles of viruses in nature^1^.

The development of whole genome sequencing has led to the elucidation of communication networks between enteric viruses and bacteria^2^. One such virus is human norovirus (HuNoV), which is a non-enveloped virus belonging to the *Caliciviridae* family that causes diarrhea and vomiting in humans and is transmitted primarily via the fecal–oral route. However, contamination of food products, such as clams and farmed oysters, has also led to HuNoV outbreaks^3^.In fact, occasional HuNoV epidemics continued to occur during the pandemic caused by severe acute respiratory syndrome coronavirus 2 despite the strict infection control measures^4,5^. The abundance of HuNoV genomes within rivers has been closely associated with the prevalence of HuNoV infection^6^. These infectious particles are assumed to reach rivers and oceans through sewage, where they are maintained in the natural environment.

Recently, sewage treatment systems have been used to monitor the circulating viruses within natural environments and communities^7^. Indeed, the safe and effective treatment of wastewater is key to preventing the spread of infectious enteric viruses. One sustainable and efficient sewage treatment strategy is the application of activated sludge^8^ containing myriad products produced by the rich microflora including Bacteroidota (Bacteroidetes), Chloroflexota (Chloroflexi), Actinomycetota (Actinobacteria), and Bacillota (Firmicutes)^9^. The abundance and activity of these phyla are positively correlated with improved pollutant removal efficiency^10^. In particular, increasing the proportion of *Bacillus* spp. in activated sludge improves sewage purification treatment capacity due to their abundant secretion of proteases^11^. Moreover, proteases produced by anaerobic bacteria reportedly reduce the abundance of poliovirus particles^2,12–14^. Additionally, *Streptomyces* spp. (Actinomycetota) produce ε-poly-l-lysine (EPL), which is a polycationic biopolymer with broad spectrum antimicrobial activity that is used as a food preservative. EPL elicits immune regulatory effects in host cells and is highly safe for human consumption^15,16^. Hence, *Caliciviridae*, including HuNoV, may also be affected by bacterial enzyme secretions; however, the associated molecular mechanisms have not been investigated.

In this study, we analyzed the effects of combining various bacterial-derived substances on the life cycle of *Caliciviridae*. Specifically, we screened the susceptibility of *Caliciviridae* to different combinations of EPL and proteases, investigated its underlying molecular mechanism, and predicted high-dimensional interactions between the anaerobic microbiome and *Caliciviridae* in the natural system.

## Results

### Serine proteases with EPL inactivate *Caliciviridae*

**Figure 1:**
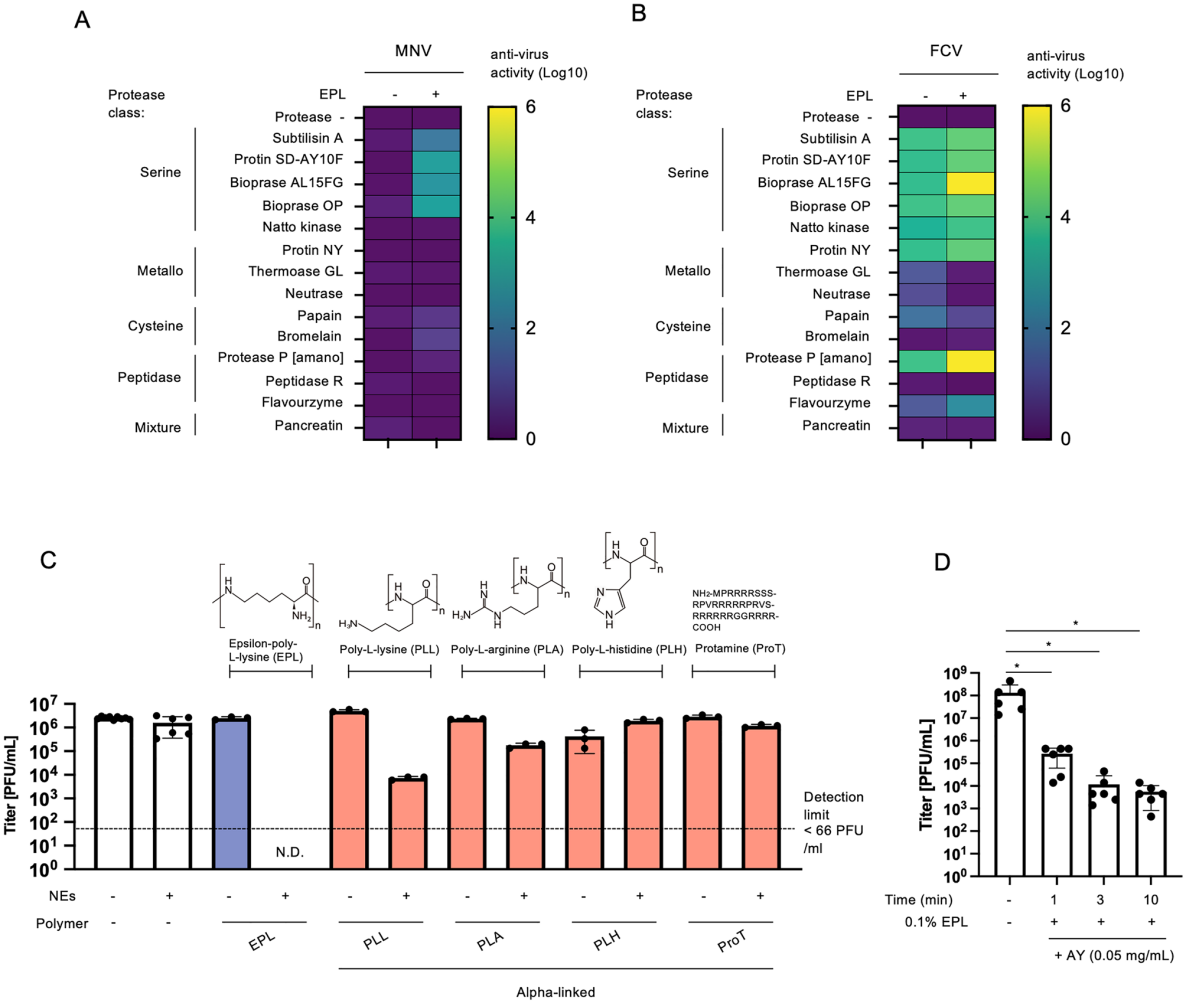
Combination of serine proteases and EPL synergistically inactivates MNV and FCV. Heat map of antiviral activity using different classes of 0.1% (*w/v*) proteases against MNV (**A**) and FCV (**B**) after incubation at 25 °C for 30 min with or without 0.1% (*w/v*) EPL. In the heatmap indicator, zero indicates no change in observation after treatment (i.e., no antiviral potential). The protease name denotes the product name. The details of the proteases are shown in Supplementary Table S1. (**C**) Plaque assay of FCV after treatment with 0.1% (*w/v*) EPL (blue) or 0.1% (*w/v*) of alpha-linked-poly-l-lysine (PLL), alpha-linked-poly-l-arginine (PLA), poly-l-histidine (PLH), or protamine (proT) (red) with NEs. Structures and sequences are shown. The bar represents mean ± SD of three independent experiments. N.D.; not detectable. The dashed line indicates the detection limit. (**D**) Time-dependency of infectious-FCV titers after treatment with AY and 0.1% (*w/v*) EPL in artificial seawater. The bar represents the mean ± SD of six independent measurements. *p < 0.05: one-way ANOVA.

First, we evaluated whether the bacterial proteases inhibited feline calicivirus (FCV) and mouse norovirus (MNV), a HuNoV surrogate (Fig. 1A,B and Table 1). We confirmed that 0.1% (*w/v*) protease did not affect with cell morphology and cell viability (Fig. S1). Among the proteases, the *Bacillaceae* serine proteases inactivated FCV and MNV in the presence of epsilon-poly-l-lysine (EPL). In contrast, metallo- and cysteine proteases and peptidases had no or weak anti-MNV and anti-FCV activities. Moreover, EPL had the most potent synergistic anti-FCV effect among the alpha-linked polycationic biopolymers, including alpha-linked poly-l-lysine (PLL), poly-l-arginine (PLA), poly-l-histidine (PLH), and protamine—an arginine-rich polypeptide—with natto extracts (NEs), comprised largely of natto kinase (NK)—a serine protease of *Bacillus subtilis* natto (Fig. 1C). Additionally, the combination of the bacterial serine protease (protin SD-AY10F; AY) produced by *Bacillus licheniformis* and EPL inhibited 99.998% of FCV infectivity in artificial seawater in a time-dependent manner (Fig. 1D).

**Table 1:** Detail of bacterial proteases used in this study.

Protease classification	Original strain	Enzyme name	Vendor
Serine	*Bacillus licheniformis*	Subtilisin A	Sigma-Aldrich (P4860)
Serine	*B. licheniformis*	Protin SD-AY10F (AY)	Amano Enzyme
Serine	*Bacillus* spp.	Bioprase AL15FG	NAGASE Co., Ltd
Serine	*Shouchella clausii*	Bioprase OP (OP)	NAGASE Co., Ltd
Serine	*Bacillus subtilis natto*	Natto kinase (NK)	FUJIFILM Wako (14-08801)
Metallo	*Bacillus amyloliquefaciens*	Protin NY	Amano enzyme
Metallo	*Geobacillus stearothermophilus*	Thermoase GL	Amano enzyme
Metallo	*B. amyloliquefaciens*	Neutrase	Novozyme
Cysteine	*Carica papaya*	Papain	Amano enzyme
Cysteine	*Ananas comosus*	Bromelain	Amano enzyme
Peptidase	*Aspergillus melleus*	Protease P [amano]	Amano enzyme
Peptidase	*Rhizopus oryzae*	Peptidase R	Amano enzyme
Peptidase	*Aspergillus oryzae*	Flavourzyme	Novozyme
Mixture	Pig pancreas	Pancreatin (mixture of amylase, trypsin, and lipase etc.)	Amano enzyme

**Figure 2:**
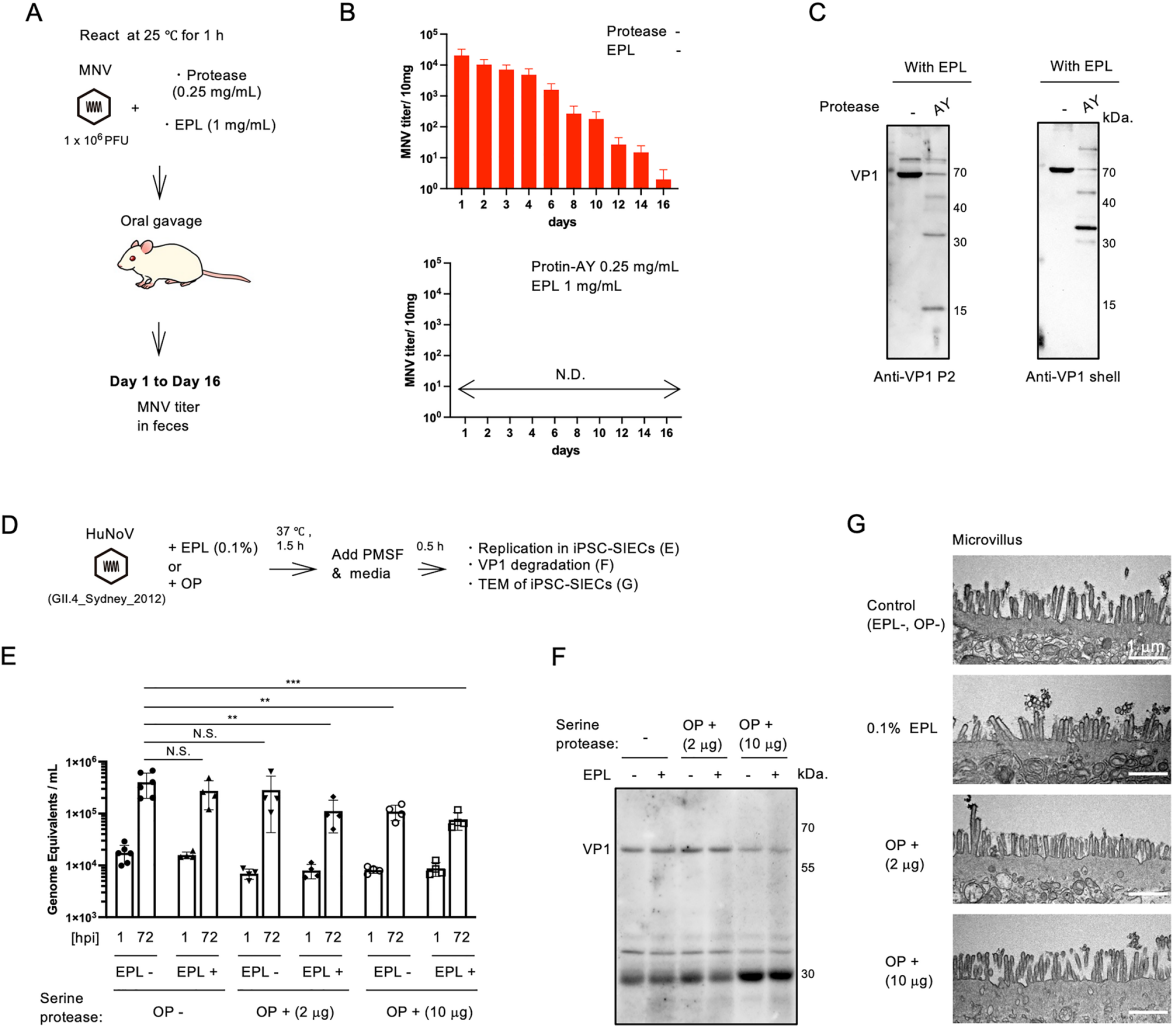
*Caliciviridae* inactivation affects replication in vivo and in human iPSC-derived SIECs. (**A**) Schematic illustration of the mouse experiments. The MNV-containing solution was administered orally after incubation with serine proteases and EPL for 1 h. (**B**) The titer of infectious viruses in feces samples was measured at the indicated period using the plaque assay. The bar represents mean ± SD, *n* = 5. N.D., not detected. (**C**) Western blotting (WB) detection of MNV VP1 (cropped) after treatment with protein SD-AY10F (AY), a serine protease, with EPL. VP1 was detected using specific antibodies against the P2 domain (right) and shell domain (left). (**D**) Schematic illustration of the human norovirus (HuNoV) experiments. (**E**) Viral replication of the HuNoV GII.4_Sydney_2012 strain at 72-h post-infection (h.p.i.) compared with that at 1 h.p.i. in iPSC-SIECs after serine protease OP treatment with or without 0.1% (*w/v*) EPL. The bar represents mean ± SD from 4–6 wells of supernatant for each condition, **p < 0.01, ***p < 0.001, N.S.: not significant; two-way ANOVA. (**F**) WB detection (cropped) of HuNoV VP1. (**G**) Microvillus image of iPSC-SIECs using TEM after treatment with OP or EPL. Note that WB images were cropped to remove irrelevant areas, and the original images are shown in Supplemental Fig. S7.

We also assessed whether the combination of serine proteases and EPL elicited inhibitory effects against infection in vivo (Fig. 2A)*.* After treatment with AY and EPL for 1 h, no infectious MNV was detected on day 1 post-virus administration (Fig. 2B). In addition, VP1—a major capsid protein^17^—was digested following treatment with EPL and AY (Fig. 2C).

To confirm the anti-viral efficacy of combined proteases and EPL against human pathogens, we assessed the replication of HuNoV GII.4_Syndeny_2012—a pandemic strain^18^ (Fig. 2D). No cell damage was observed after adding serine protease inhibitor while efficient VP1 digestion was observed using Bioprase OP (OP) (Fig. S2). Thus, we used OP for HuNoV GII. 4. Results showed a significant dose-dependent reduction in replication following OP and EPL treatment of induced pluripotent stem cell-derived small intestinal epithelial cells (iPSC-SIECs)^19^ (Fig. 2E). That is, a 71% decrease in intact VP1 was observed after OP (10 μg) and EPL treatment (Fig. 2F). However, no change was observed in microvilli structure or cell surface morphology in iPSC-SIEC after EPL or OP treatment (Fig. 2G). Thus, combining *Bacillaceae* serine proteases and EPL can inactive *Caliciviridae* viruses, including HuNoV.

### Conformational changes are induced in the capsid structure by combined serine proteases and EPL

**Figure 3:**
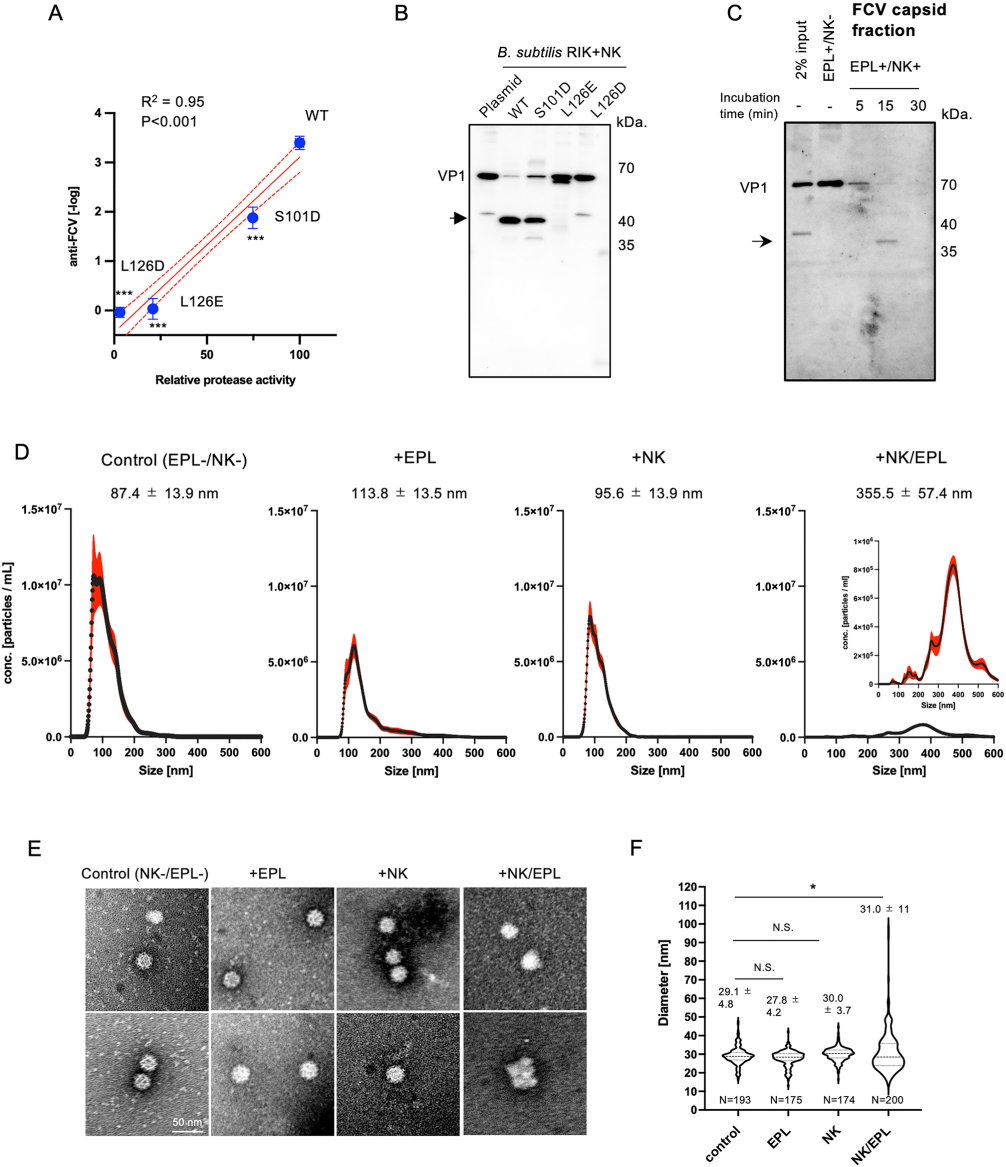
NK and EPL combination induces morphological changes in FCV particles. (**A**) Correlation between anti-FCV activity and protease activity of NK and its mutants. The anti-FCV activity was calculated from infectious-FCV titers after treatment with 0.1% (*v/v*) culture supernatant of wild type (WT) or its mutants and 0.1% (*w/v*) EPL. Protease activity of NK WT was set to 100%. The red solid and dashed lines indicate the regression line and 95% confidence interval, respectively. Data are represented as mean ± SD of four independent experiments. ***p < 0.001 versus anti-FCV activity of WT; Student’s *t*-test. (**B**) Western blot (WB) analysis (cropped) of FCV after treatment with supernatants from the NK mutants with EPL. (**C**) FCV VP1 degradation kinetics of capsid particles. After treatment with NK and EPL at the indicated time, the capsid was analyzed in the eluate in fraction no. 17 (approximately 5000 kDa) via size-exclusion chromatography. VP1 was detected via WB with rabbit anti-FCV VP1 (cropped). (**D**) Purified FCV particles were incubated with or without 0.1% (*w/v*) NK and 0.1% (*w/v*) EPL at 25 °C for 30 min. Particle size distribution was measured using nanoparticle tracking analysis. Data are shown as mean ± SEM (solid red line). Three independent quintuplicate measurements; ***p < 0.01, ****p < 0.001 versus without NK and EPL as controls; one-way ANOVA. (**E**,**F**) Representative electron microscopy images (**E**) and violin plots (**F**) of the FCV diameter in two independent experiments. N indicates the number of FCV particles analyzed. *p < 0.05, N.S.: not significant; one-way ANOVA. Note that WB images were cropped to remove irrelevant areas, and the original images are shown in supplemental Fig. S7.

Using biochemical approaches, we investigated the mechanism by which serine proteases in combination with EPL reduced the infectious FCV titer. To this end, we generated a deficient mutant of NK protease activity at the S3 site^20^ (Fig. 3A). Upon treating FCV with a supernatant containing NK or its mutants from *B. subtilis* RIK 1285 strains, anti-FCV activity was observed, which further corroborated with the degradation of VP1 observed in the western blotting. The anti-FCV potency and NK protease activity in the mutants were strongly correlated; the L126D and L126E mutants lost their FCV inactivation ability even in the presence of EPL. The NK protease activity was consistent with VP1 degradation (Fig. 3B). In addition, PMSF—a serine protease inhibitor—negated the anti-FCV activity and VP1 degradation of NEs and EPL (Fig. S3). Furthermore, to verify the destruction of the capsid structure following interaction with the protease and EPL, intact viral particles were isolated via size exclusion chromatography and detected by western blotting using anti-FCV VP1. The degradation of VP1 was time-dependent following NK treatment (Fig. 3C, Fig. S4); a digested VP1 fragment of approximately 37 kDa was detected after 15 min of incubation. Furthermore, nanoparticle tracking analysis showed that EPL or NK alone induced a slight particle number reduction but showed a diameter distribution comparable to that of the purified FCV particle (Fig. 3D). In contrast, compared with the control FCV, co-incubation with NK and EPL resulted in a 6.92-fold decrease in particle number and a further dramatic shift in diameter to approximately 355 nm (Fig. 3D). In addition, transmission electron microcopy analysis showed that compared with untreated 30 nm FCV particles, treatment with NK with EPL caused viral particle distortion and aggregation (Fig. 3E). Similarly, the diameter of FCV treated with NK and EPL was widely distributed compared with that of untreated FCV, FCV treated with EPL, or FCV treated with NK alone (Fig. 3F). Therefore, serine proteases in combination with EPL might contribute to the induction of morphological changes in viral particles through capsid protein digestion.

### EPL induces conformational changes in the capsid to a protease-sensitive form

Based on the unique and pivotal synergistic effect of EPL (Fig. 1), we hypothesized three possible functions of EPL that could enhance *Caliciviridae* sensitivity to serine proteases. First, EPL accelerates the proteolytic activity of serine proteases. Second, EPL acts as a buffering agent for alkaline pH because it is a highly cationic biopolymer (pKa = 9)^21^; the optimal pH for serine proteases is generally 8–10. Third, EPL induces a conformational change in the capsid, making it more sensitive to serine proteases.

Initially, no change in the proteolytic activity of NK was observed irrespective of EPL treatment (Fig. S5A). Although sterilized water containing NEs had a neutral pH of 7.0, the EPL suspension shifted to an alkaline pH between 7.5 and 8, dependent on the EPL volume (Fig. S5B). The FCV titer was measured in pH-adjusted HEPES buffer in NEs without EPL, which clearly decreased at pH 8, consistent with the observed VP1 degradation (Fig. S5C,D). These results suggest that FCV inactivation is highly dependent on pH conditions as alkaline pH accelerated the serine protease-mediated FCV inactivation. This observation supports the second hypothesis.

**Figure 4:**
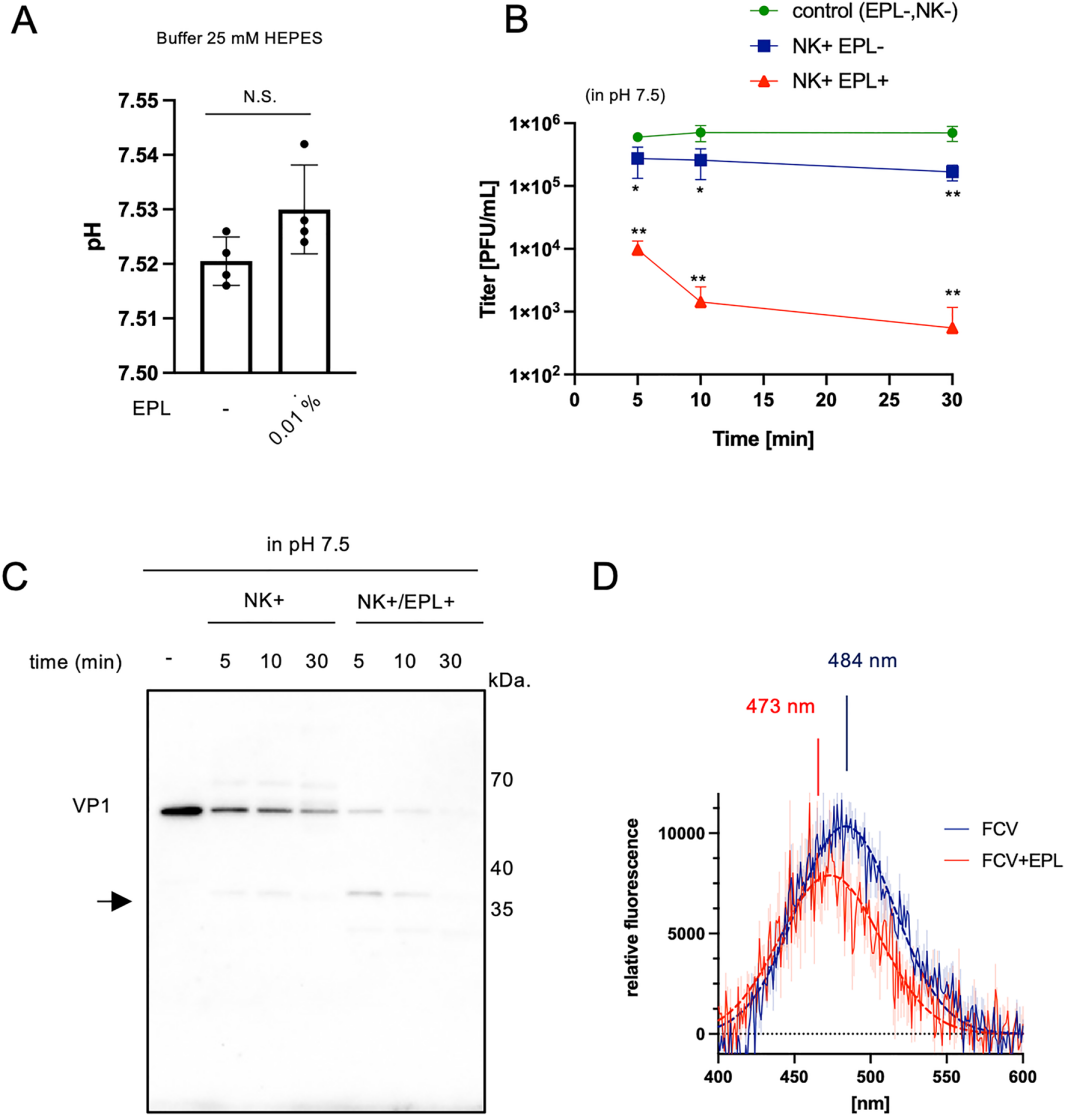
EPL promotes serine protease-dependent viral inactivation through alkaline pH shifting and conformational changes. (**A**) pH measurement in 25 mM HEPES–NaOH at pH 7.5 with 0.01% (*w/v*) EPL. N.S.; not significant. (**B**) Infectious-FCV titer after treatment with or without 0.1% (*w/v*) NK and 0.01% (*w/v*) EPL in 25 mM HEPES–NaOH at pH 7.5. Data are shown as mean ± SD of three independent experiments. *p < 0.05; **p < 0.01 versus control at each indicated time point; Student’s *t*-test. (**C**) Western blot (WB) detection of FCV VP1 after treatment with combined NK and EPL for the indicated period. The arrow indicates a degraded fragment. WB image was cropped to remove irrelevant areas, and the original images are shown in supplemental Fig. S7. (**D**) Fluorescence spectra of purified FCV with (red) or without (blue) EPL obtained using ANS, a hydrophobic probe. The maximum wavelength is indicated. Data are shown as mean ± SD of three independent experiments.

Finally, we investigated the third possibility that EPL induces capsid conformational changes using a 25-mM HEPES containing buffer with pH fixed at 7.5. Exogenous addition of 0.01% (*w/v*) of EPL did not affect the pH (Fig. 4A). Infectious FCVs were detected after 30 min of incubation with NK, which was consistent with anti-FCV potency using NEs at pH 7.5 (Fig. S5C). Whereas the combination with EPL in a pH-fixed condition markedly reduced infectivity within 5 min (Fig. 4B). VP1 was immediately digested by NK combined with EPL by the 5 min time point (Fig. 4C). To detect structural changes in the capsid with high sensitivity, we used the hydrophobic probe 8-anilinonaphthalene-1-sulfonate (ANS), and quantified the fluorescence intensity. The peak intensity of the purified FCV capsid without EPL was measured at a wavelength of 484 nm (Fig. 4D, blue). Upon incubation with EPL, the peak shifted to 10 nm, and the intensity decreased (Fig. 4D, red). These results indicate that EPL induces conformational changes in the capsid to a protease-sensitive form, which proves the third hypothesis.

### Combined serine proteases and EPL suppress RNA release from the capsid by cleaving specific sites in the P2 and S domains

**Figure 5:**
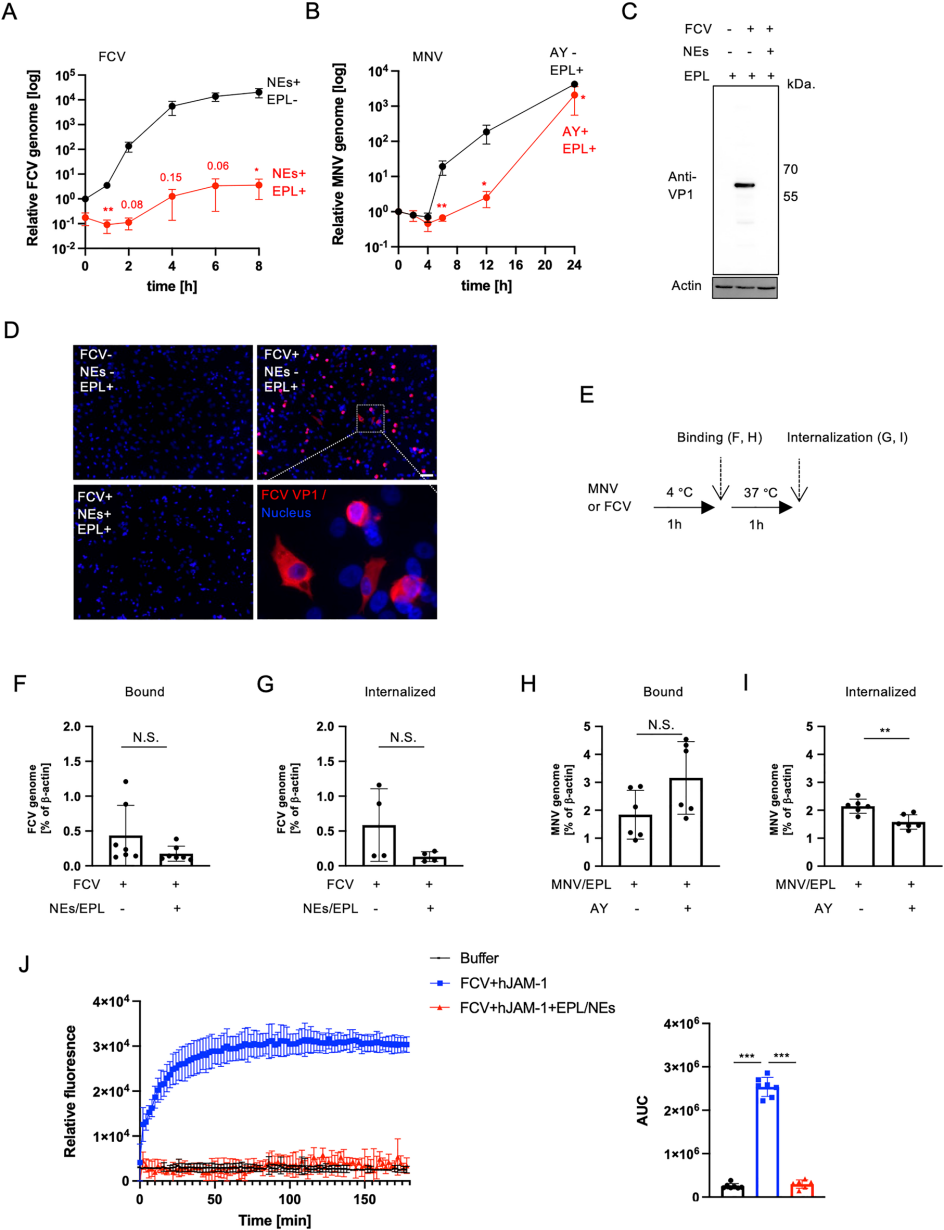
Combined serine proteases and EPL impair RNA release from the FCV capsid. (**A**) FCV genome replication kinetics after treatment with 0.1% (*w/v*) EPL (black), 0.1% (*w/v*) NEs, and 0.1% (*w/v*) EPL combination (red) for 30 min at 25 °C. Data are shown as mean ± SEM of three independent experiments. *p < 0.05 versus indication at each time point; Student’s *t*-test. (**B**) MNV genome replication kinetics after treatment with 0.1% (*w/v*) AY protease (red) without protease (black) with 0.1% (*w/v*) EPL present. Data are shown as mean ± SD of four independent experiments. *p < 0.05, **p < 0.01 versus indication at each time point without protease; Student’s *t*-test. (**C**,**D**) Western blotting (WB: cropped) and immunofluorescence staining (**D**) of FCV VP1 (red) expression at 4 h post-infection with indicated combinations. White bars indicate 50 μm. Raw data of the western are shown in supplemental Fig. S7. (**E**) Schematic representation of binding and internalization assays involving incubation at 4 °C and additional incubation at 37 °C, respectively. (**F to I**) Genome detection of absorbed FCV on the cell surface (**F**) and internalized FCV in the cytoplasm (**G**) with or without 0.1% (*w/v*) NEs and 0.1% (*w/v*) EPL treatment. The bar represents mean ± SD of seven (**F**) or four (**G**) independent experiments., N.S.:not significant; Student’s *t*-test. Genome detection of absorbed MNV on the cell surface (**H**) and internalized FCV in the cytoplasm (**I**) after treatment with or without 0.1% (*w/v*) AY and 0.1% (*w/v*) EPL at 25 °C for 30 min. The bar represents the mean ± SD of five independent experiments. **p < 0.01, N.S.: not significant; Student’s *t*-test. (**J**) Kinetic analysis of fluorescence intensity (left) and area under the curve (right) of RNA released from purified FCV with hJAM-1. RNA release kinetics without treatment (blue lines) and with 0.1% (*w/v*) EPL and 0.1% (*w/v*) NE combination treatment (red line). The fluorescence intensity of the buffer (negative control, without FCV and other proteins) is indicated by a black line. Data are shown as mean ± SD of seven independent measurements. ****p < 0.001; one-way ANOVA.

To better understand the virological inactivation mechanisms of the serine proteases in combination with EPL, we investigated the replication kinetics of *Caliciviridae*. We chose the NEs containing NK and AY for FCV and MNV, respectively, to identify a common inactivation mechanism for the serine protease class. In addition, we compared different reaction conditions for FCV and MNV (i.e. only NEs treatment as a control for FCV, and EPL treatment as a control for MNV). Due to differences in the time to onset of cytopathic effect (CPE) causing viral replication in the cell, we quantified the viral genome up to 8 h and 24 h for FCV and MNV, respectively. NEs and AY with EPL impaired FCV and MNV replication (Fig. 5A,B). Owing to inactivation by the serine protease and EPL combination, no FCV VP1 was observed via western blotting and immunofluorescence analysis at 4 h post-infection (Fig. 5C,D). These results suggest that the proteolysis of capsid proteins could affect any of the first three steps of the viral life cycle (i.e., attachment, entry, and uncoating). Therefore, virus attachment and internalization were next quantified by detecting the viral genome after being incubated with cells at 4 °C for 1 h and recovered at 37 °C for 1 h (Fig. 5E). Neither NEs nor AY with EPL affected virus attachment. Similarly, the internalization of FCV after NEs and EPL treatment were comparable (Fig. 5F–I). In contrast, although internalized MNV was significantly reduced after AY and EPL treatment, there were slight changes compared to a reduction of infectious virus particles (Fig. 1A), suggesting serine proteases and EPL combination would affect the uncoating step. Thus, we analyzed the uncoating ability of FCV capsids in vitro. The RNA genome is released from the capsid upon conformational changes in capsid proteins such as VP1 and VP2 under acidic conditions during virus receptor binding^22^. The RNA fluorescent probe detected the real-time kinetics of genome release from the capsid during hJAM-1 receptor binding (Fig. 5J), indicating that the RNA genome was released. In contrast, the fluorescence intensity and area under the curve after treatment with NEs and EPL were comparable to those for the control buffer alone (Fig. 5J).

**Figure 6:**
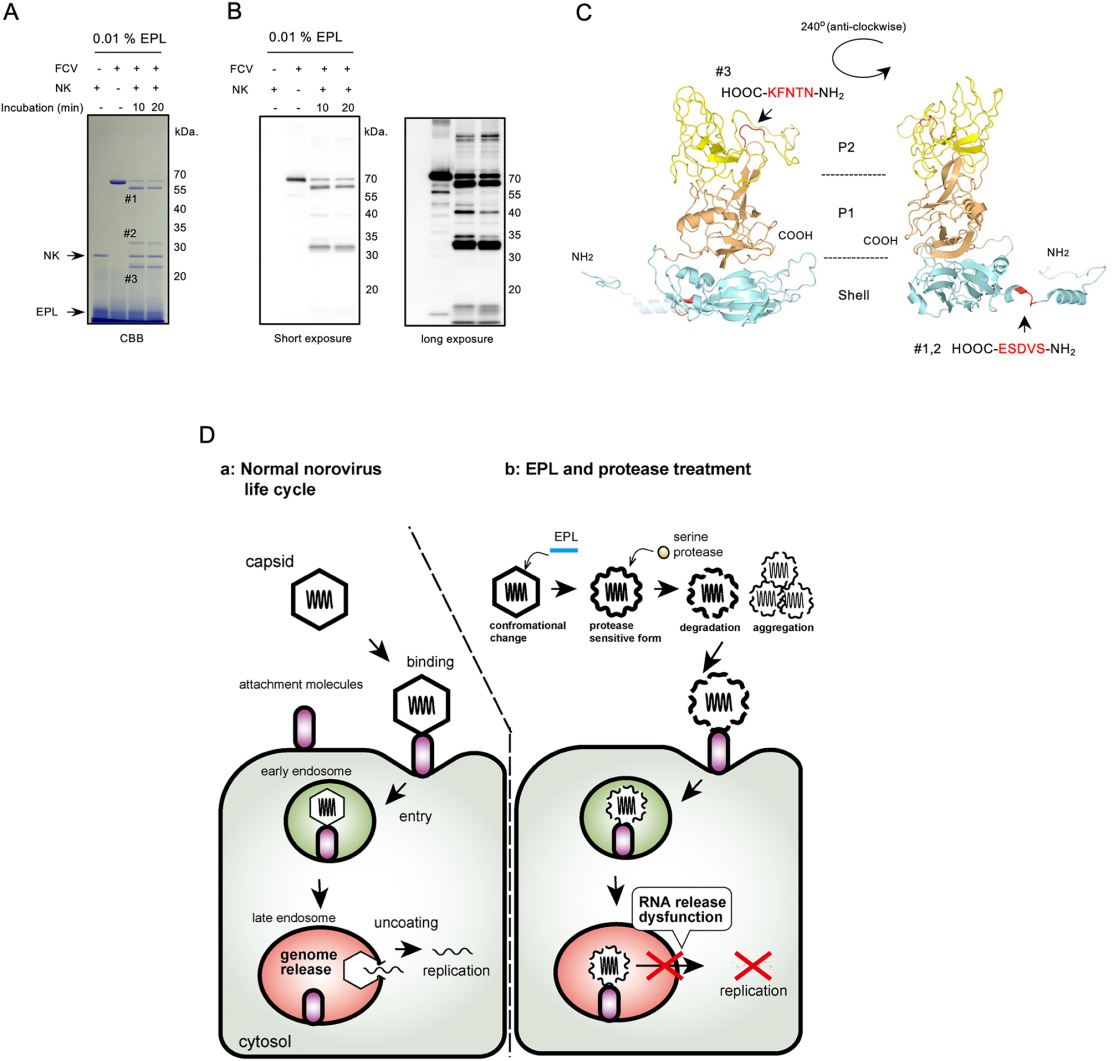
NK digests a specific region of FCV in the P2 and shell domains. (**A**,**B**) Purified FCV were incubated with 0.01% (*w/v*) EPL and 0.025% (*w/v*) NK at 25 °C for up to 20 min in 25 mM HEPES–NaOH at pH 7.4. The proteolytic profile was visualized using Coomassie brilliant blue (CBB) staining (**A**) and western blotting (WB) (**B**) with a rabbit anti-FCV VP1 antibody, which recognizes the shell and partially recognizes the P1 and P2 domains (details are shown in Fig. S6). #1 to #3 in (**A**) indicates the fragments analyzed using N-terminal amino acid sequencing. WB images were cropped to remove irrelevant areas, and the original images are shown in supplemental Fig. S7. (**C**) Mapping of the N-terminal amino acid sequence of fragments (#1 to #3) on the 3D structure of FCV VP1 proteins. The shell, P1, and P2 domains are denoted in light cyan, light orange, and light yellow, respectively. Red indicates the NK cutting site. The right panel is 240° counterclockwise from the left panel. (**D**) Graphic summary of this study: mechanism of *Caliciviridae* inactivation by a serine protease in combination with EPL. The left panel shows the general life cycle of *Caliciviridae*. First, the virus is absorbed onto the cell surface via binding to a specific receptor. Second, the virus enters endocytic pathways. Third, in the uncoating step, the RNA genome is released from the capsid following conformational changes induced by acidic shock via endosome maturation. Fourth, genome replication occurs, and viral proteins are synthesized in the cytoplasm. The bacterial serine protein-dependent *Caliciviridae* inactivation with EPL. First, EPL (blue bar) induces conformational changes in the capsid, increasing its sensitivity to serine proteases such as NK, AY, or OP. Secondly, the capsid particles are partially cut by the proteases, resulting in cracking or aggregation. Third, the capsid partially enters the cytoplasm after cell absorption. Fourth, the RNA genome released during the uncoating step becomes dysfunctional via proteolytic cutting (i.e., a partial cutting of VP1 or capsid aggregation by a serine protease). Fifth, *Caliciviridae* replication is suppressed.

Finally, to identify the position of the protease cleavage in the capsid, N-terminal amino acid sequencing of the fragments was performed (Fig. 6A). SDS-PAGE analysis showed that proteolysis by NK with EPL produced three fragments, named #1 (~ 55 kDa), #2 (> 30 kDa), and #3 (20–30 kDa). The anti-FCV VP1 antibody was created from the recombinant protein containing the S domain and a portion of the P1/P2 domains (1–326 amino acids) and recognized degradation products #1 and #2 (Figs. 6B and S6A). In contrast, fragment #3 was not detected via western blotting. Based on the N-terminal amino acid sequencing results, #3 was identified as NTNFK in the P2 domain (Figs. 6 and S6A). In contrast, #1 and #2 had the same sequence as SVDSE in the S domain. These results suggest that the FCV VP1 fragments #1–3 were aligned (Fig. S6B). To map the #1–3 sequence to the 3D structure of FCV VP1 (Figs. 6C and S6C–F), the #3 of the NK cleavage site was located near the flexible loop in the P2 domain. Hence, capsid cleavage by serine proteases with EPL inhibited RNA genome release from the virus particles.

## Discussion

In this study, we demonstrated how combinations of bacterial secretions affect *Caliciviridae* viruses, including the HuNoV life cycle. The most effective combinations of serine proteases and EPL are produced by the *Bacillaceae and Streptomyces* families, belonging to the Bacillota and Actinomycetota phyla, respectively. These phyla are abundant in sewage sludge^23^ and are related to sewage purification efficacy^24^. Based on the results of the current study, the mechanism by which combined serine proteases and EPL are applicative for removing *Caliciviridae* from the environment primarily involves the cleavage of viral capsid proteins and the subsequent release dysfunction of the RNA genome. Ultimately, contact between *Caliciviridae*, Bacillota, and Actinomycetota may influence the *Caliciviridae* replication cycle in nature.

*Streptomyces* cationic EPL interacts with negatively charged molecules, such as the phosphate group in the gram-negative bacterial outer-membrane lipopolysaccharide or bacterial and fungal cellular membrane phospholipids, resulting in membrane disruption in a detergent-like manner^15,21,25^. Considering the electrostatic surface potentials on FCV, the capsid has a negatively charged pocket between the A–B and C–C dimers located in the NK cleavage site on the P2 domain (Fig. S6E,F), suggesting that “straight-shaped” EPL might become inserted into the pocket, triggering surface conformational changes to a serine protease-sensitive form.

*Caliciviridae* viruses enter cells by binding to viral receptors such as CD300lf for MNV and fJAM-1 for FCV^22,26,27^. Subsequently, the RNA genome is released into the cytoplasm in its uncoated form owing to the rearrangement of the capsid structure^22^. Within the *Caliciviridae* life cycle, the RNA-releasing ability was significantly inhibited by the combination of serine proteases and EPL, indicating an apparent delay in genome replication. Structural analysis revealed that the NK cleavage site on the P2 domain closely localized to the loop at the 438–448 position, which was released by binding JAM-1, the driving force of the conformational changes in the capsid structure (Fig. S6C,D)^22^. Among the cationic amino acid polymers, only EPL showed an alkaline shift. These results correlated with the chemical properties, as the pKa of EPL was 9^21^, and alkaline pH induces a change in the capsid structure^28^.

The mechanism of the synergistic effects of EPL and bacterial serine proteases on *Caliciviridae* infectivity is as follows: EPL maintains the optimal alkaline pH for the serine protease, promoting a conformational change in the capsid to a protease-sensitive form; the serine proteases then degrade the capsid, preventing release of the RNA genome into the cytoplasm. Overall, the EPL and the serine proteases interfere with *Caliciviridae* replication (Fig. 6D).

Agricultural soils are known to inactivate enteric viruses^29^. EPL is produced by the abundant *Streptomyces* in the soil as well as by various other bacteria in soil and seawater^16^. Additionally, the *Bacillaceae* family produces numerous serine proteases and are abundant in soil. Bacterial extracellular compounds such as lipopolysaccharides and peptidoglycans save the enterovirus infectivity^30^. Enterovirus, including enterovirus 71 (EV71) and Coxsachievirus-A9 virus, are inactivated by bacterial serine proteases in lake water^2^. Similarly, enveloped and non-enveloped viruses are inactivated by bacterial proteases^12–14^. For example, the serine protease alcalase can inactivate rotavirus, a member of the *Reoviridae* family, that causes diarrhea^13^. Meanwhile, pronase—a protease mixture produced by *Streptomyces griseus—*inhibits Coxsackievirus A9 replication^14^. Hence, based on our results as well as those of previous studies, *Calicivirus*, including HuNoV, might be inactivated by contact with EPL and serine proteases in the soil and seawater.

The vesicle coating of HuNoV facilitates its stability in feces, enabling its fecal–oral transmission^31^. Additionally, the protective vesicles support multiple rounds of replication within salivary gland cells^32^. Therefore, contact with vesicle-coated viral particle is an important transmission^33^. Although the combination of EPL and OP effectively inactivated the pandemic GII.4_Sydney_2012 strain in stool, the efficiency and VP1 degradation were moderate. This was due to the serine protease not being able to directly access the vesicle membrane-enwrapped capsid. In fact, the VLPs of HuNoV were digested by the serine protease regardless of the majority (GII.4) and minority (GI.1) circulating genotypes^18,34^ (data not shown). Hence, HuNoV protects itself against bacterial serine proteases and EPL by enwrapping virus particles in the vesicle membrane, reflective of a survival strategy adopted by *Caliciviridae*. In addition, OP has been known to be produced from *Shouchella clausiI* (formerly *Bacillus clausii*)*,* which already has been used as a probiotic; administration of *S. clausii* improves acute gastroenteric symptoms such as diarrhea^35^. Thus, we believe that either OP or *S. clausii* administration with EPL would improve the acute gastroenteric symptoms caused by enteric viruses.

Further analysis is required to elucidate how *Caliciviridae* are circulated or degraded in nature and, to develop a decontamination approach using proteases, such as sodium hypochlorite, known as the most effective disinfectant to date.

Finally, our findings advance the understanding of high-dimensional interactions between *Caliciviridae* and bacterial products such as EPL and serine proteases. However, the symbiotic interkingdom interactions between viruses and bacteria in nature comprise an extremely elaborate, complicated, and adaptable system that includes metabolites and secretions. Accordingly, further investigation is required to elucidate this sophisticated system.

## Methods

### Reagents

Poly-l-arginine (PLA; P4663) and poly-l-histidine (PLH; P2534) were purchased from Sigma-Aldrich (St. Louis, MO, USA). Protamine was obtained from FUJIFILM Wako Pure Chemical Corporation (Osaka, Japan). A 25% (*w/v*) solution of EPL was provided by JNC Corporation (Tokyo, Japan). Alpha-linked poly-l-lysine (PLL; 3075) was purchased from the Peptide Institute, Inc. (Osaka, Japan). The detailed proteases are presented in Table 1. Artificial seawater powder (product name is UMIJIO; 553161) was obtained from KAMIHATA Fish Industries Ltd. (Himeji, Japan). All antibodies are listed in Table S1.

### Virus and culture

Feline calicivirus (FCV; F9 strain, VR-782: taxonomy ID, 11981 in National Center for Biotechnology Information) and CRFK (a *Felis catus* kidney cell line) were obtained from the American Type Culture Collection (ATCC; Manassas, VA, USA) and cultured in Dulbecco’s modified Eagle’s medium (DMEM) supplemented with 10% (*v/v*) fetal bovine serum (FBS), 100 U/mL penicillin, and 100 μg/mL streptomycin (P/S). The mouse norovirus (MNV) culture protocol has been previously reported^36^. All cells were cultured at 37 °C and 5% CO_2_. FCV was purified according to the protocol described by Conley et al.^22^.

### Measurement of antiviral activity using plaque assay and VP1 degradation

For plaque assay of FCV, 3.0 × 10^5^ CRFK cells were seeded in 12-well plates at 24 h prior. FCV, 0.1% (*v/v*) NEs, 0.1% (*w/v*) NK, or 0.1% (*v/v*) overnight culture supernatants of *B. subtilis natto* were diluted in sterile water or 1 × PBS (−) in the presence or absence of 0.1% (*w/v*) EPL. After 30 min of incubation at 25 °C, the reaction was arrested by adding an equal volume of medium containing 10% (*v/v*) FBS, and tenfold serial dilutions were prepared. Dilutions (300 μL) were inoculated at 37 °C. After 1 h of incubation, the virus-containing medium was removed, and 1 mL of DMEM supplemented with 1.1% (*w/v)* carboxy methyl cellulose, 2% (*v/v*) FBS, and P/S was overlaid and incubated at 37 °C for 2 days. Before staining, the cells were fixed by adding 10% (*v/v*) formalin supplemented with 0.15 M NaCl for at least 2 h, and plaques were stained and enumerated as described previously^37^. A plaque assay of the MNV was performed according to a previous report^36^. The anti-viral activity was calculated by subtraction from the control (i.e., without protease). To check for VP1 degradation, PMSF (Sigma-Aldrich), a serine protease inhibitor, was added, and incubated for 10 min. Subsequently, 6 × SDS-sample buffer (Nacalai tesque, Kyoto, Japan) was added and boiled at 100 °C for 2 min. The titer shown in Fig. 1D was obtained using median tissue culture infectious dose (TCID50) and converted to plaque forming unit (PFU) using the formula, PFU = 0.7 × TCID50^38^.

### Purification of natto extracts

Natto extracts (NEs) containing natto peptides from fermented natto soybeans (Yamada Foods Co., Ltd.; Akita, Japan) were prepared as described previously^39^. Natto soybeans were soaked in 3 volumes of 10 mM Tris–HCl (pH 7.4) and mixed at 4 °C overnight. The supernatant was collected via centrifugation at 5000×*g* for 10 min. An equal volume of ethanol was added, followed by incubation at 4 °C for 1 h with shaking to remove polyglutamine. Subsequently, the supernatant was collected by centrifugation at 5000×*g* for 10 min. Ethanol was removed using an evaporator. The NEs were then fractionated using ammonium sulfate precipitation (30–50% (*w/v*)). After overnight dialysis with 10 mM Tris–HCl (pH 7.4), the samples were concentrated by freeze-drying.

### Mice

All mice used in the study were 6-week-old female BALB/c mice (Japan SLC, Hamamatsu, Japan) housed under a 12-h/12-h light/dark cycle. This study was approved by the Animal Care Committee of the Chubu University (permission number: 3010060). All mouse experiments were carried out according to relevant guidelines and regulations. Furthermore, the results were reported in accordance with ARRIVE guidelines. The MNVs (1 × 10^6^ plaque forming units (PFU)/mL) were incubated with serine protease and EPL at 25 °C for 1 h. Next, 0.2 mL of a 100-fold diluted suspension with 1 × PBS (−) containing 2 × 10^3^ PFU/mL MNV was orally administered to the mice using an oral sonde. Stool samples were collected daily throughout the study period. After the final collection, all mice (*n* = 20) were sacrificed via intraperitoneal injection of excess sodium pentobarbital (150 mg/kg of mice). The infectious MNV titer in feces was determined per a previously-reported plaque assay protocol using 1.5% (*w/v*) SeaPlaque™ agarose (Lonza, Rockland, ME, USA)^40^.

### Human norovirus preparation

HuNoV-containing stool samples were dissolved at 10% (*w/v*) in 1 × PBS (−) and filtered twice using a 0.22-μm filter via centrifugation at 12,000×*g* for 5 min at 4 °C each. Aliquots were stored at − 80 °C until use. Viral genomes were purified using the QIAamp Viral RNA Mini kit (Qiagen, Venlo, The Netherlands) following the manufacturer’s instructions. Genotyping was performed using Norovirus Typing Tool Ver. 2.0^41^. The copy number was quantified using Takara qPCR Norovirus (GI/GII) typing kit ver.2 (Takara Bio, Kusatsu, Japan) and normalized as genome equivalents (GE) per ml.

### Human small intestinal epithelial cells

Induced pluripotent stem cell-derived small intestinal epithelial cells (iPSC-SIECs; 1 × 10^5^) (FUJIFILM Wako) were seeded onto Matrigel (Corning; Corning, NY, USA; #354230)-coated 96-well plates (Corning; 9102) using a seeding medium (FUJIFILM Wako) and incubated overnight at 37 °C in a CO_2_ incubator. Cells were then cultured in a culture medium (FUJIFILM Wako), which was replenished every 2 days. At 11 days post-seeding, cells were preincubated with 0.5 mM glycochenodeoxycholic acid sodium salt (GCDCA) for 1.5 h. HuNoV (GII.4 [P16]_sydeny_2012) filtrates (1.43 × 10^6^ GE) were incubated with 0.1% (*w/v*) EPL or 2 or 10 μg of serine protease OP at 37 °C for 1.5 h in a 20-μL reaction volume. They were then incubated with 3 volumes of 1.125 mM phenylmethylsulfonyl fluoride (PMSF)-supplemented culture medium for 30 min. iPSC-SIECs were then infected with 1.07 × 10^6^ GE of HuNoV at 37 °C for 1 h. Cells were washed twice with 200 μL of culture medium. Additionally washing twice with 150 μL, the medium (totally 300 μL) were collected for viral genome quantification at 1 h.p.i. Furthermore, cells were incubated with 150 μL of culture media with 0.5 mM GCDCA for 72 h, and the culture supernatants, as well as 150 μL of washing media, were collected (72 h.p.i). Purification and quantification were conducted using the QIAamp Viral RNA mini kit and Takara qPCR Norovirus (GI/GII) typing kit ver.2, respectively.

### Expression of Natto kinase mutants

The cDNA of the Natto kinase (NK) gene was amplified via PCR from the isolated *B. subtilis natto*. Point mutations (S101D, L126D and L126E) were constructed using NEBuilder HiFi DNA assembly (New England Biolabs, Ipswich, MA, USA). Wild type (WT) and mutants were expressed using the *B. subtilis* secretory protein expression system (Takara Bio). A single colony was cultured in a trypticase soy broth with 0.6% yeast extract (TSBYE) media supplemented with 10 μg/mL kanamycin at 37 °C for 2 days with shaking; the supernatant was filtered using a 0.2-μm filter (Merck Millipore, Burlington, MA, USA). Aliquots were stored at − 30 °C until use.

### Protease activity measurement

The protease activity of NK mutants was measured using an Amplite Universal Fluorometric Protease Activity Assay kit (AAT Bioquest, Sunnyvale, CA, USA). Kinetics of protease activity was observed up to 1 h. Protease activity was calculated from the area under the curve of kinetics. The protease activity of the NK in the presence of EPL (Fig. 3A) was measured according to a previously described protocol using the Infinite M200 Pro microplate reader (TECAN, Mannedorf, Switzerland)^42^.

### Size-exclusion chromatography

First, 600 μL of the FCV mixture was reacted with 0.1% (*w/v*) EPL and NK at 25 °C for up to 30 min. To prevent protein degradation by the protease activity of NK during preparation, PMSF was added, followed by incubation for 10 min before boiling. Next, 500 μL of the mixture was loaded onto a Superose 6 10/30 column equilibrated with 0.15 M NaCl and 20 mM Tris–HCl (pH 7.4). The eluents were fractionated as 500-μL samples at a flow rate of 0.6 mL/min. The particle size distribution was calibrated using a gel filtration standard (Bio-Rad, Hercules, CA, USA).

### FCV particle number and size measurement

The purified FCV particles were diluted in 25 mM HEPES–NaOH (pH 7.4) and passed through a 0.22-μm filter. One milliliter of the mixture containing 0.5 × 10^6^ PFU of FCV with or without 0.01% (*w/v*) EPL and/or NK was incubated at 25 °C for 30 min. The particles were then immediately examined using a NanoSight NS300; the number and size of the viral particles were analyzed using Nanoparticle Tracking Analysis software (Malvern Panalytical, Worcestershire, UK).

### Electron microscopy

After reaction with NK and EPL, purified FCV was placed on a carbon-coated grid (ELS-C10: Okenshoji Co. Ltd., Tokyo, Japan). After incubation for 1 min, 4% (*w/v*) uranyl acetate was added to the suspensions, followed by gentle mixing and incubation for 30 s. Images were observed via transmission electron microscopy (TEM) using the JEM-1400 system (JEOL, Tokyo, Japan). The FCV size was calculated using Adobe Photoshop software (Adobe, San Jose, CA, USA).

### Measurement of conformational changes in the FCV capsid

8-Anilinonaphthalene-1-sulfonate (ANS) was dissolved in dimethyl sulfoxide (DMSO). The following reactions were carried out in black 96-well plates. The purified FCV (4.7 μg) was incubated with or without 0.01% (*w/v*) EPL in 25 mM HEPES–NaOH (pH 7.4) at 25 °C for 20 min. Subsequently, ANS (final, 20 μM) was added and incubated at 25 °C for 5 min. The fluorescence of the samples was monitored at an excitation wavelength of 365 nm and emission wavelength of 400–600 nm using a TECAN M1000 Pro system. The final spectra were obtained after subtracting the background spectra (i.e., buffer only or EPL containing only ANS).

### Binding and internalization assay and measurement of genome replication kinetics

To assess cell surface binding, the FCV or MNV samples were first treated with proteases and incubated at 4 °C for 1 h with shaking. To quantify the internalized virus genome, the cells were incubated at 37 °C for 1 h, trypsinized, and washed three times with cold 1 × PBS (−). To monitor genome replication after recovery at 37 °C, the cells were collected at the indicated time points after washing with cold 1 × PBS (−). Total RNA was purified using the RNeasy Plus kit (Qiagen). Next, cDNA was synthesized using the SuperScript IV VILO system (Thermo Fisher Scientific, Waltham, MA, USA). The FCV genome was detected via SYBR Green-based Quantitative PCR (qPCR) using the KOD SYBR qPCR Mix kit (TOYOBO, Osaka, Japan). The MNV genome was quantified via Taqman probe-based qPCR using the THUNDERBIRD Probe qPCR Mix (TOYOBO). qPCR was performed using LightCycler 480 System II (Roche, Basel, Switzerland). The FCV and MNV genome levels were normalized to the percentage of expression of the reference gene, β-actin. The primer details are presented in Table S2.

### Immunofluorescence assay

After 4 h of infection, cells were cultured on a collagen-coated 12-mm cover glass (AGC TECHNO GLASS Co., Ltd., Shizuoka, Japan) and fixed using 4% (*w/v*) paraformaldehyde at 25 °C for 15 min. Specimens were treated with 5% (*v/v*) normal donkey serum (Abcam, Cambridge, UK) and 0.3% (*v/v*) Triton X-100 in 1 × PBS (−) for 1 h after washing with 1 × PBS (−). The anti-FCV VP1 antibody was diluted with 1% (*w/v*) BSA and 0.3% Triton X-100 in 1 × PBS (−) and incubated at 4 °C overnight. After rinsing with 1 × PBS (−), the specimens were incubated with Alexa Fluor 594 IgG at 25 °C for 1 h. Finally, the specimens were embedded in ProLong Gold Antifade reagent with 4′,6-diamidino-2-phenylindole (Cell Signal Technology, Danvers, MA, USA) after washing with 1 × PBS (−) six times. Fluorescent images were obtained using a BZ-X700 (KEYENCE, Osaka, Japan) and cropped with Adobe Photoshop software (Adobe).

### RNA release assay

The RNA release assay was modified from a previously reported protocol^22^. Briefly, recombinant human JAM-1 (hJAM-1; Sino Biological, Inc., Beijing, China) was dissolved in 5% (*v/v*) glycerol, 0.15 M NaCl, and 10 mM Tris–HCl (pH 7.4) and stored at − 80 °C until use. Next, 5 μL of purified FCV (approximately 1.7 × 10^6^ PFU) was mixed with 5 μL of NEs and EPL (final concentration, 0.063% (*w/v*) each). After incubation at 25 °C for 30 min, the FCV samples were incubated with 4 μL of hJAM-1 (2 μg) at 25 °C for 10 min. Finally, a mixture containing 85 μl of 0.5 M sodium acetate buffer (pH 6.0) and 1 μl of 0.5 mM SYTO9™ Green dye was added to monitor RNA release. The fluorescence of the samples was measured every 2 min for 180 min using the TECAN M1000 Pro system. The amount of RNA released was calculated by subtracting the values obtained for the corresponding samples without hJAM-1 from the values obtained with hJAM-1. The area under the curve was calculated using the GraphPad Prism 9 software (GraphPad Software, San Diego, CA, USA).

### N-terminal amino acid sequencing

Purified FCV (12.4 μg) was incubated with 0.01% (*w/v*) EPL and 0.025% (*w/v*) NK at 25 °C for 20 min in 25 mM HEPES–NaOH (pH 7.4). Next, PMSF (final 5 mM) was added and incubated on ice for 5 min to stop the protease reaction. Subsequently, 2.4 μg (for N-terminal sequencing) and 0.05 μg (for western blotting) of FCV were separated via 12.5% SDS-PAGE. After transferring the proteins to an immobilon-P^SQ^ membrane (Merck Millipore), the bands were visualized after staining with 0.1% (*w/v*) Brilliant Blue R250 in 50% (*v/v*) methanol for 1 min. The membranes were then rinsed with 50% (*v/v*) methanol and dried. The N-terminal amino acid sequences were analyzed using Procise492HT (Thermo Fisher Scientific), and the identified sequences were mapped onto the FCV VP1 structure of the F9 strain (PDB ID: 6GSH)^22^ using Waals software (Altif Laboratories, Inc., Tokyo, Japan).

### Statistics and reproducibility

Descriptive statistics were calculated using Prism v.9 or Microsoft Excel (Microsoft Corporation, Redmond, WA, USA). Differences with P-values < 0.05 were considered statistically significant using one-way ANOVA or Student’s t-test (two-tailed). No statistical method was used to predetermine the sample size. Experiments were generally reproducible. All analyzed data are presented as the mean ± standard deviation (SD) except the data of the replication kinetics analysis (shown in Fig. 5A) and nanoparticle tracking analysis, which are shown as the mean ± standard error of the mean (SEM).

### Supplementary Information

Supplementary Information.

## Data Availability

All raw data obtained in this study are stored strictly within the laboratory of Sapporo Medical University in accordance with the guidelines. The data that support the findings of this article and its supplementary information are available from the corresponding author (N.O.) upon reasonable request.
