# Supplementary material for: Bacillaceae serine proteases and Streptomyces epsilon-poly-l-lysine synergistically inactivate Caliciviridae by inhibiting RNA genome release
Source: Sci Rep. 2024 Jul 2;14:15181. doi: 10.1038/s41598-024-65963-9 (PMC11219925; doi:10.1038/s41598-024-65963-9)
Supplement: Supplementary file 1 — Supplementary Information. [file 41598_2024_65963_MOESM1_ESM.pdf]

Supplemental Information

Bacillaceae **serine proteases and** ***Streptomyces*** **epsilon-poly-L-lysine synergistically inactivate** ***Caliciviridae*** **by inhibiting RNA genome release**

Soh Yamamoto[1], Noriko Ogasawara[1,2,*], Yuka Sudo-Yokoyama[1], Sachiko Sato[1], Nozomu Takata[3], Nana Yokota[4], Tomomi Nakano[4], Kyoko Hayashi[5], Akira Takasawa[6], Mayumi Endo[1], Masako Hinatsu[4], Keitaro Yoshida[1], Toyotaka Sato[7,8,9], Satoshi Takahashi[10], Kenichi Takano[2], Takashi Kojima[11], Jun Hiraki[4], and Shin-ich Yokota[1]

[1] Department of Microbiology, [2] Department of Otolaryngology -Head and Neck Surgery, [10] Department of Infection Control and Laboratory Medicine, [11] Department of Cell Science, Sapporo Medical University School of Medicine, Sapporo 060-8556, Japan

[3] Center for Vascular and Developmental Biology, Feinberg Cardiovascular and Renal Research Institute, Feinberg School of Medicine, Northwestern University, Chicago IL 60611, USA

[4] Yokohama R&D Center, JNC Corporation, Yokohama 236-8605, Japan

[5] College of Life and Health Sciences, Chubu University, Kasugai 487-8501, Japan

[6] Department of Pathology, Asahikawa Medical University, Asahikawa 078-8510, Japan

[7] Laboratory of Veterinary Hygiene, Faculty of Veterinary Medicine, Hokkaido University, Sapporo 060-0818, Japan

[8] Graduate School of Infectious Diseases, Hokkaido University, Sapporo 060-0818, Japan

[9] One Health Research Center, Hokkaido University, Sapporo 060-0818, Japan

*Correspondence: Noriko Ogasawara (ogasawara.n@sapmed.ac.jp); Sapporo Medical University School of Medicine, Sapporo 060-8556, Japan

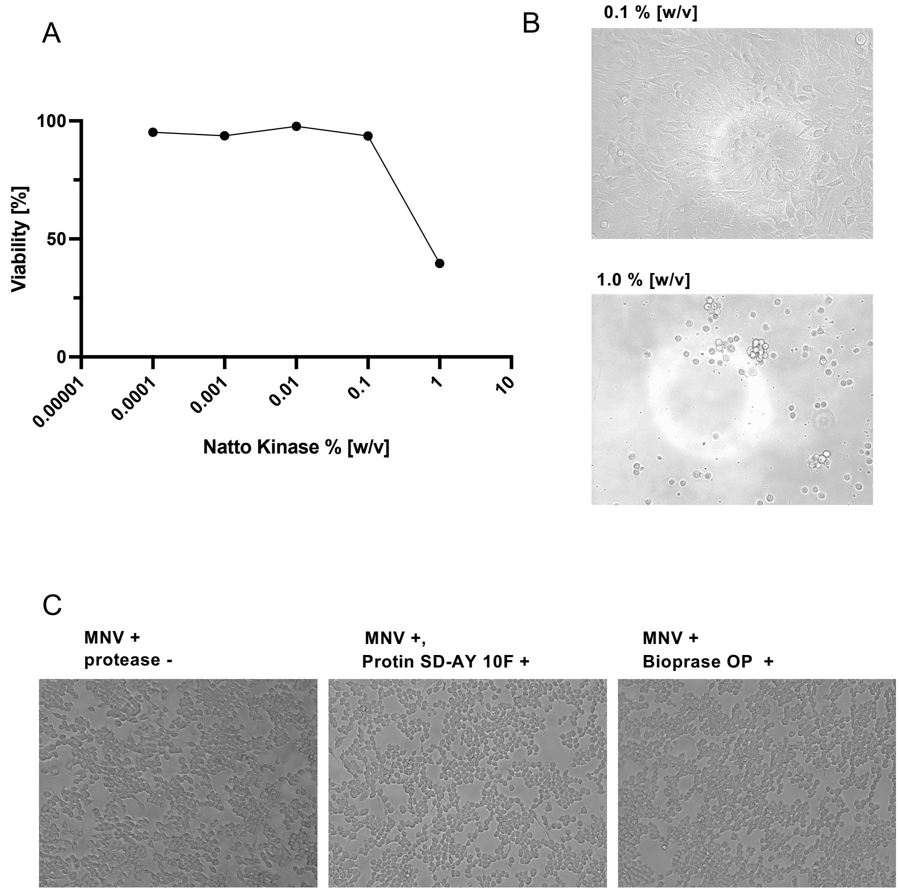

Fig. S1 Cell viability and morphological changes after serine protease treatment.
(A) MTT assay of CRFK cells after natto kinase treatment overnight at 37 °C. (B) morphology of CRFK cells after 0.1 or 1.0 % (*w/v*) natto kinase treatment overnight at 37 °C. (C) morphology of RAW 264.7 cells after 0.1 (*w/v*) protin SD-AY 10F or bioprase OP treatment for 2 h at 37 °C.

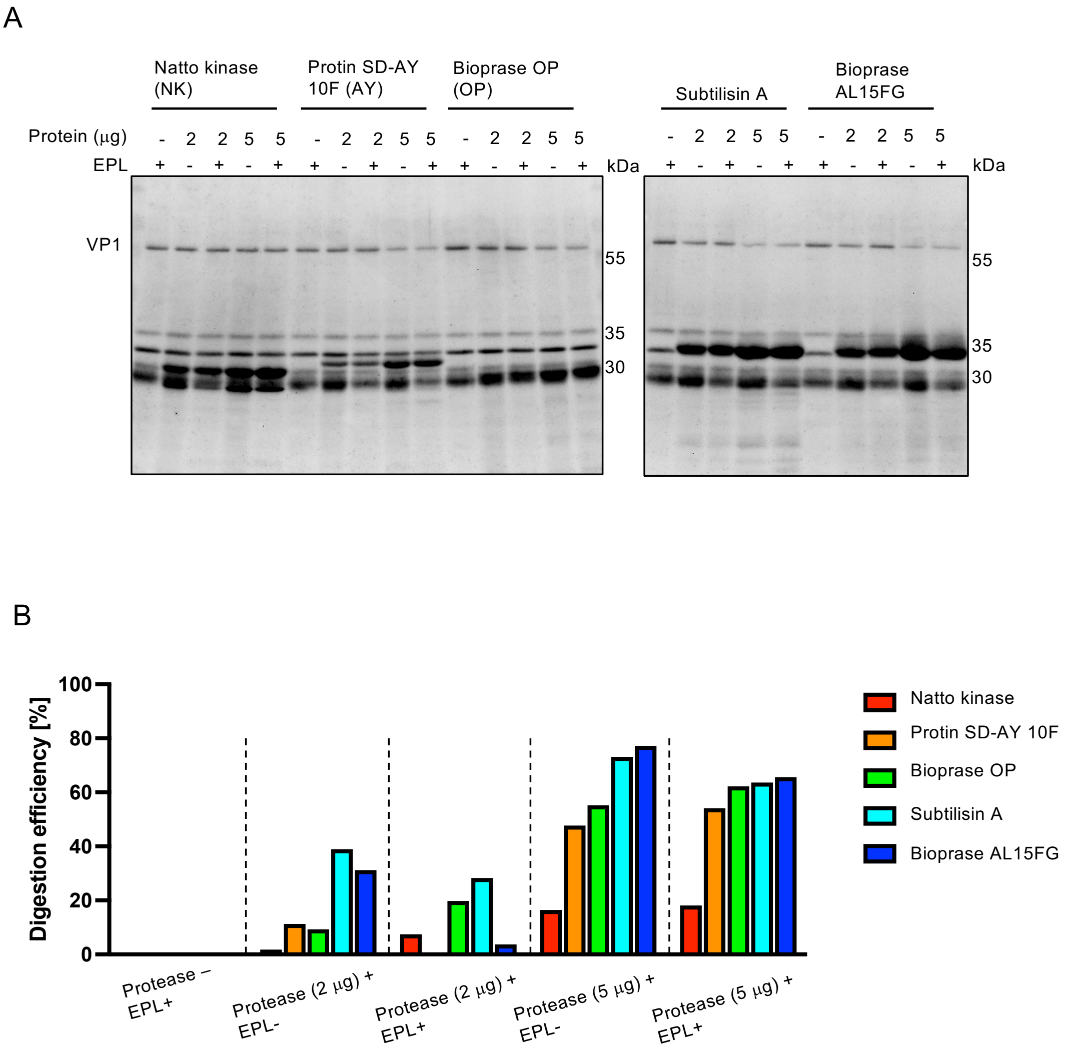

Fig. S2 VP1 digestion screening of serine proteases against human norovirus (HuNoV) GII. 4. Human fecal specimen containing HuNoV GII. 4 was incubated with 0.1 % (*w/v)* serine proteases indicated and 0.1 % (*w/v)* EPL for 1 h at 37 °C, then PMSF (final 2 mM) was added and additionally incubated for 10 min at 25 °C. (A) intact HuNoV VP1 proteins were detected via western blotting (WB). (B) Quantitative of undigested VP1. VP1 in the absence of protease and in the presence of EPL was set to 100%. Note that the WB image was cropped to remove irrelevant areas, and the original images are shown in supplemental Fig. S8.

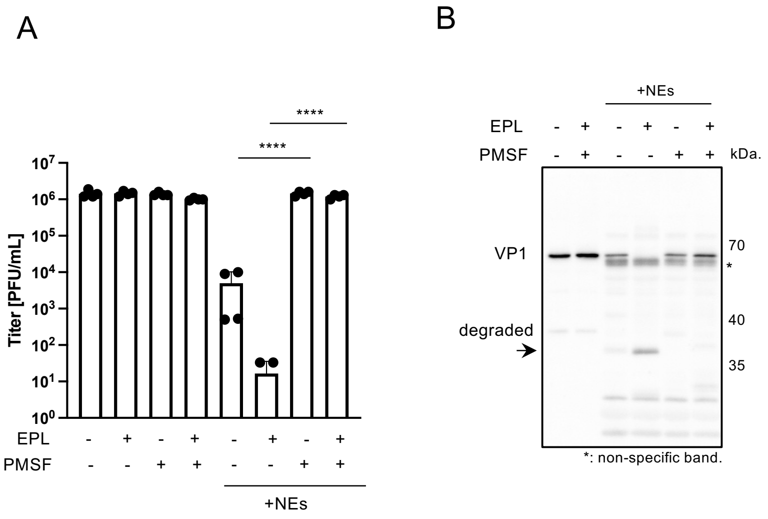

Fig. S3 PMSF, a serine protease inhibitor, negates the anti-FCV potency of NEs and EPL.

(A) Plaque assay of FCV after 0.1% (*w/v*) NEs and 0.1% (*w/v*) EPL treatment with or without PMSF for 30 min at 25 °C. The bar represents the mean ± SD of four independent experiments, ****p < 0.0001; one-way ANOVA. (B) FCV VP1 detection via western blotting (WB) after incubation with NEs and EPL with or without PMSF. Asterisks indicate non-specific bands. Note that the WB image was cropped to remove irrelevant areas, and the original images are shown in supplemental Fig. S8.

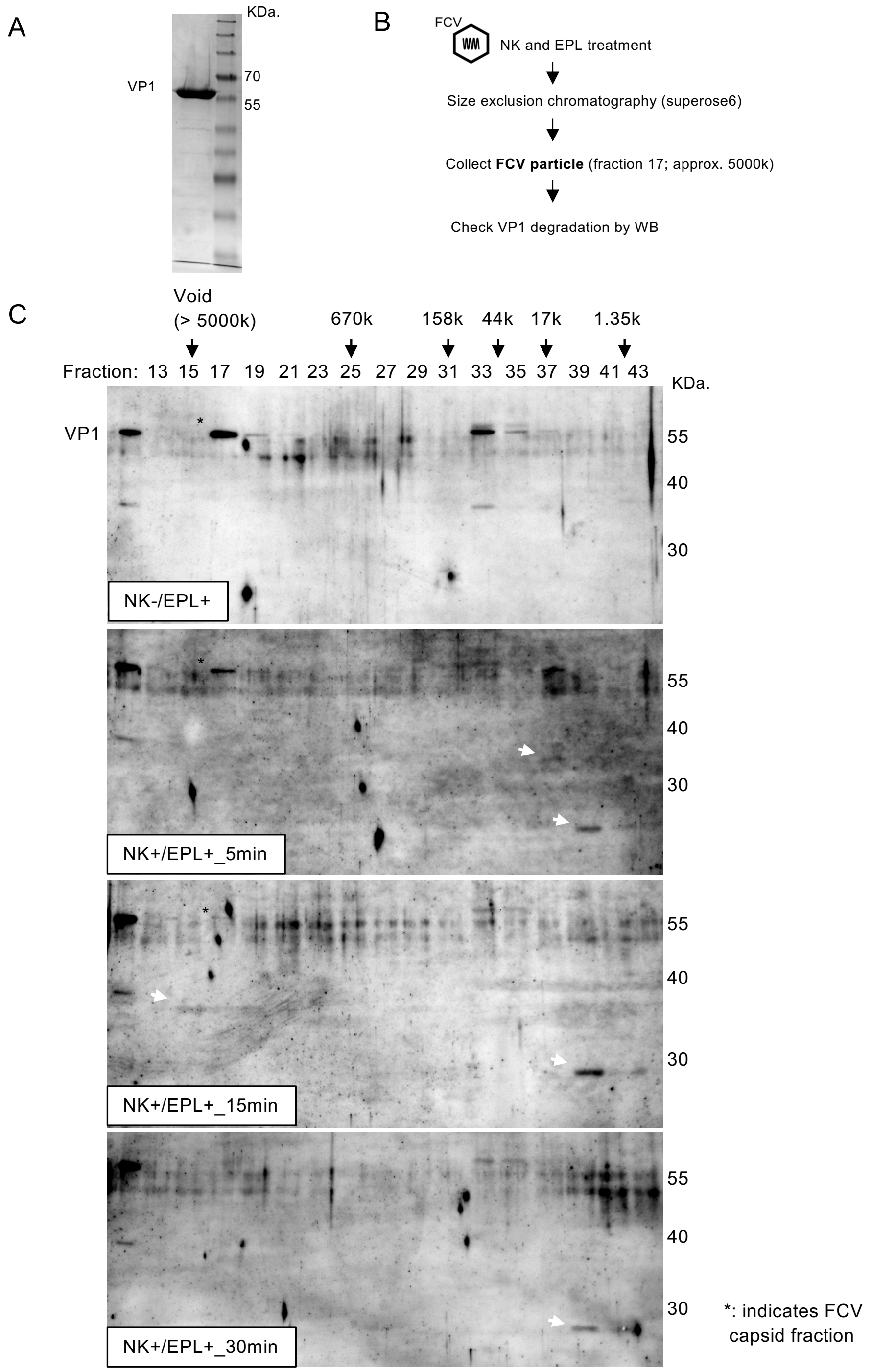

Fig. S4 Size-exclusion chromatography for FCV after treatment with natto kinase (NK) and EPL. (A) SDS-PAGE (cropped) of purified FCV particles (5 μg/lane), indicating > 99% purity. (B) Isolation strategy after treatment. The FCV capsid in the virus-amplified stock (before purification) was eluted at approximately 5,000 kDa, and the monomer or dimer VP1 was eluted in fraction 33 between 158 and 44 kDa. (C) Western blotting analysis of FCV VP1 in each eluate. White arrow indicates the degradation of the VP1 protein. The asterisk denotes the VP1 protein in the capsid. Please note that the SDS-PAGE image was cropped to remove irrelevant areas, and the original images are shown in supplemental Fig. S8.

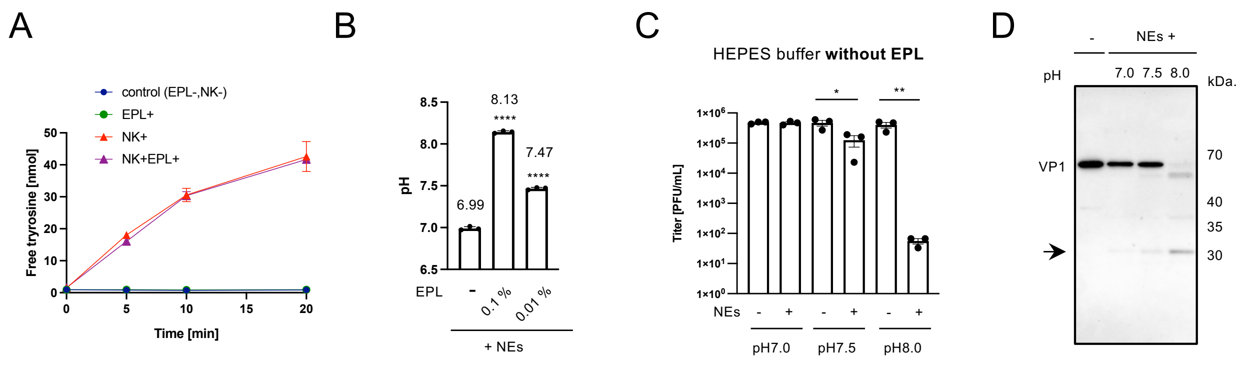

Fig. S5 Characterization of EPL property for protease activity and pH shift. (A) Proteolytic kinetics of NK with or without EPL. Data are shown as mean ± SD of four independent measurements. (B) pH shift after mixing 0.01% or 0.1% (*w/v*) EPL with 0.1% (*w/v*) NEs. The bar represents mean ± SD of four independent measurements. (C and D) Infectious-FCV titer (C) and western blotting (WB) (cropped) (D) detection of FCV VP1 after reaction with NEs in 25 mM HEPES-NaOH buffer adjusted from pH 7 to final pH 8 without EPL. The bar represents mean ± SD of three independent measurements. *$p < 0.05$; **$p < 0.01$; Student's *t*-test. Please note that the WB image was cropped to remove irrelevant areas, and the original images are shown in supplemental Fig. S8.

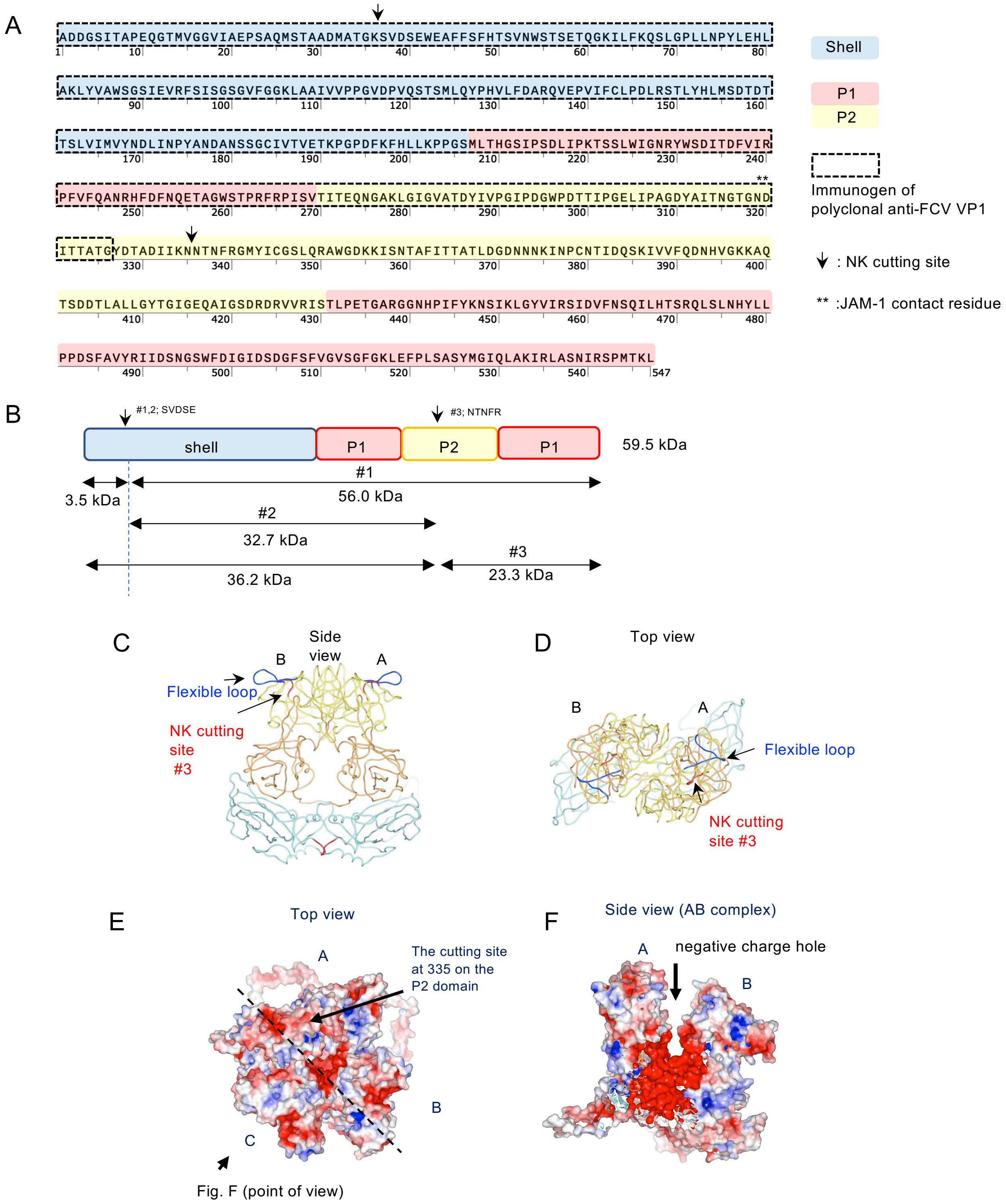

Fig. S6 Details of the primary sequence of FCV VP1 and its natto kinase (NK) cutting site. (A) Primary amino acid sequence of the FCV VP1 protein. The shell, P1, and P2 domains are indicated in blue, red, and yellow, respectively. Dashed boxes indicate the region of the immunogen used for antibody production. Arrows indicate the NK cutting site obtained using N-terminal amino acid sequencing. (B) Prediction of FCV VP1 fragments using N-terminal sequencing. (C and D) Ribbon diagrams of the VP1 A-B dimer from side view (C) and top view (D). Red and blue indicate the NK

cutting site and the loop lifted by JAM-1 binding. The electrostatic surface potential data (3m8l-ABC) of FCV F5 were obtained from the eF-site in the Protein Database Bank Japan (PDBj). The graphic image was illustrated using the Waals software. (E and F) Electrostatic surface potential details of the FCV VP1 ABC complex from top view (E) and side view (F). A side view is shown along the dashed lines in (E). The viewpoint of position C is shown in (E) as an arrow.

Fig. 2C Anti-VP1 P2

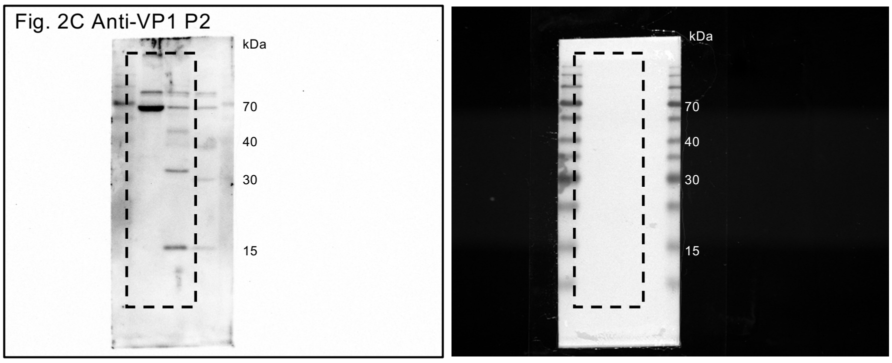

Fig. 2C Anti-VP1 shell

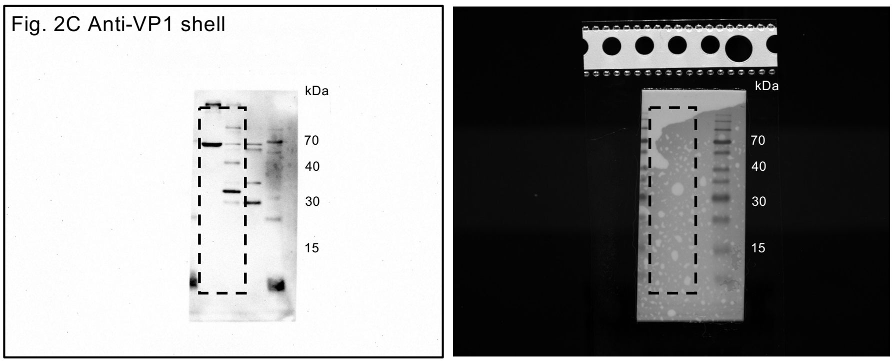

Fig. 2F

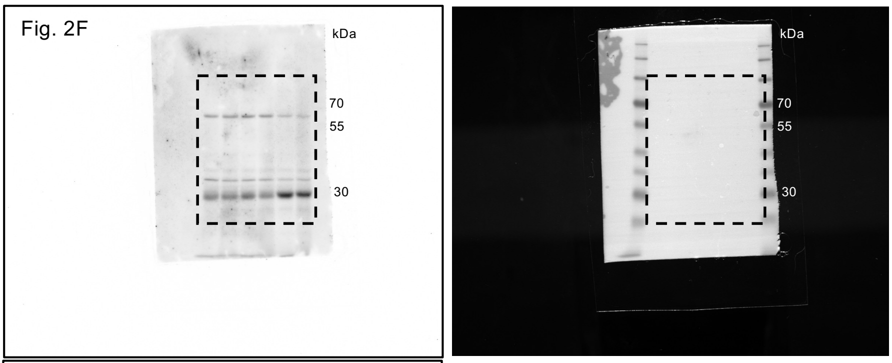

Fig. 3B

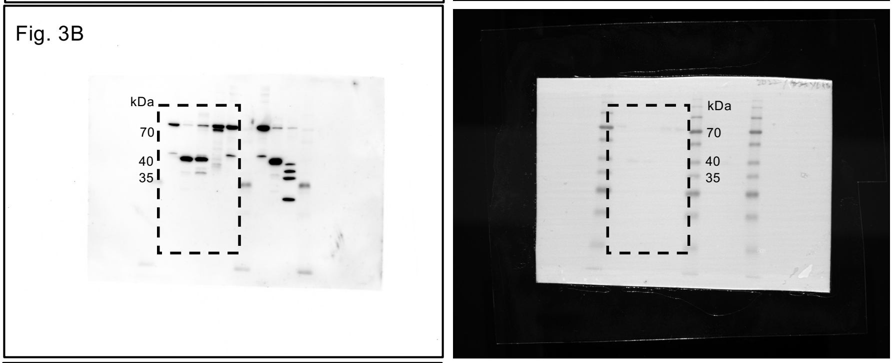

Fig. 3C

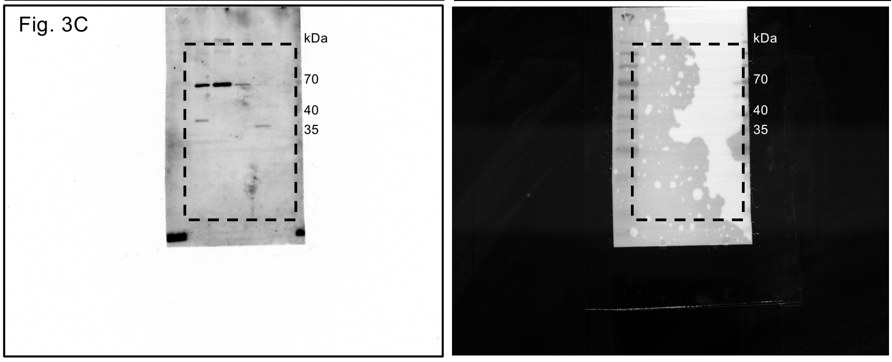

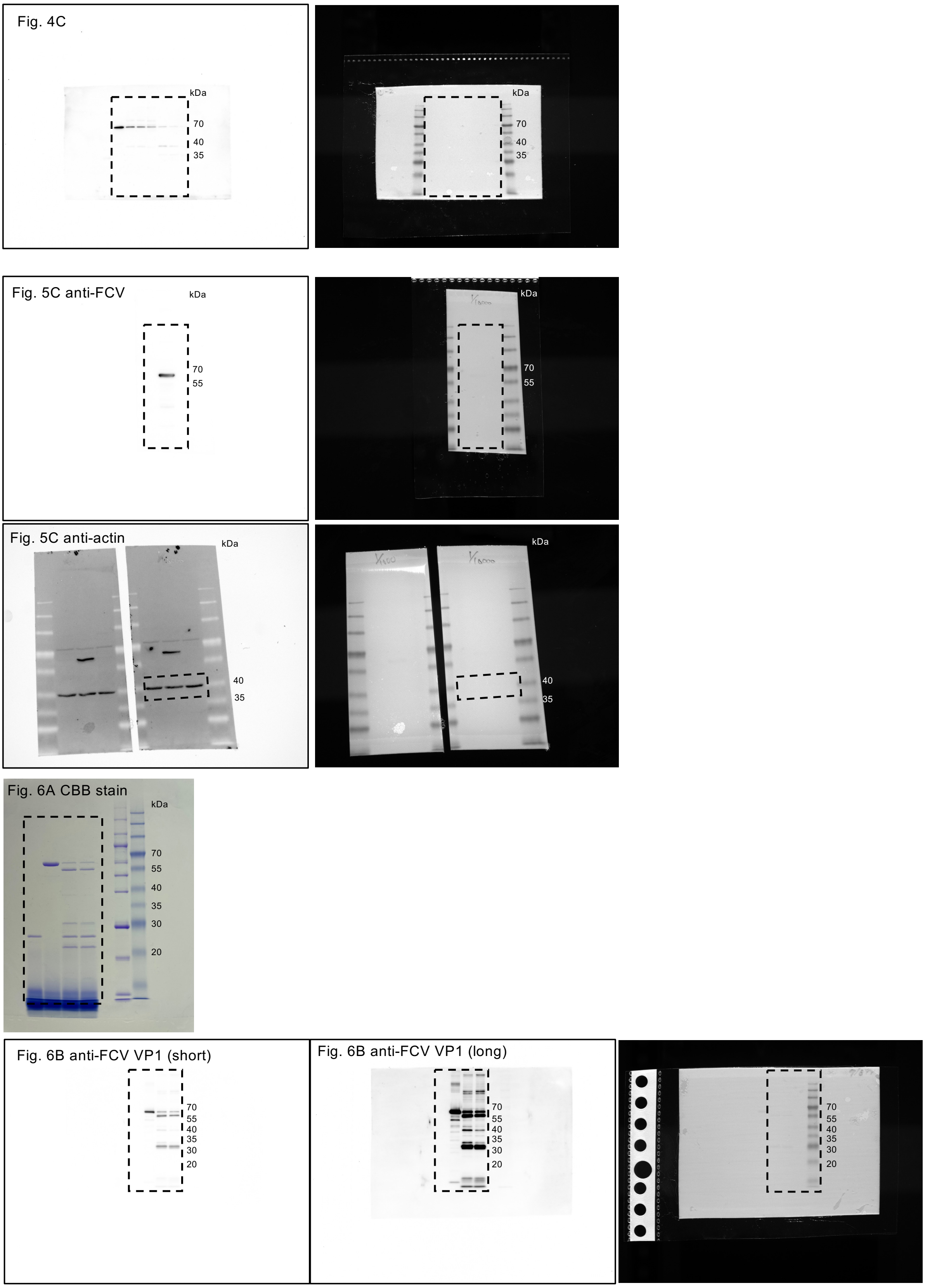

Supplemental Figure S7. Uncropped western blotting images of Figures 2C, 2F, 3B, 3C, 4C, 5C and 6B, and uncropped CBB-stained SDS-PAGE gel image of Fig. 6A.

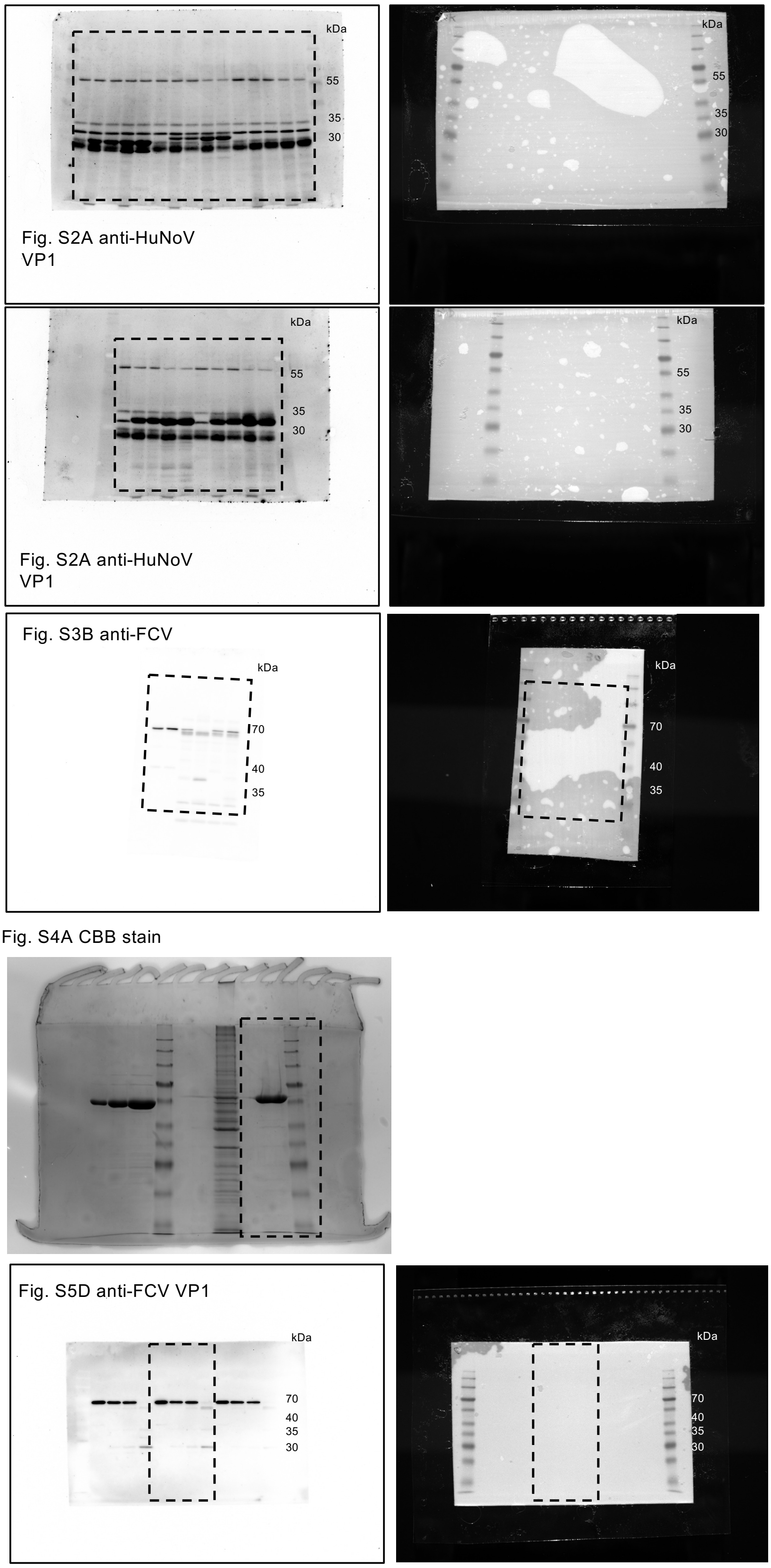

Supplemental Figure S8. Uncropped western blotting images of Figures S2A, S3B and S5D, and uncropped CBB-stained SDS-PAGE gel image of Figure S4A**.**

Table S1 Information of antibodies used in this study.

Name	Clone	Vendor
Mouse anti-HuNoV capsid antibody	NVGC-01	Bio Academia (Osaka, Japan)
Rabbit anti-MNV VP1 P2 domain antibody	This study*.<br>Generated using purified recombinant protein VP1 P2 domain (278–415 amino acids)	Cosmo Bio Co., Ltd. (Tokyo, Japan)
Rabbit anti-MNV VP1 shell domain antibody	This study*.<br>Generated using purified recombinant protein MNV VP1 shell domain (1–277 AA)	Cosmo Bio Co., Ltd. (Tokyo, Japan)
Rabbit anti-FCV VP1 antibody	This study*.<br>Generated using purified recombinant protein FCV VP1	Cosmo Bio Co., Ltd. (Tokyo, Japan)

*These anti-MNV VP1 P2 domain, shell domain, and anti-FCV VP1 antibodies were purified using Protein A Sepharose chromatography.

Table S2 Information of primers for real-time PCR

Primer name	Sequence (5'–3')	Purpose	Detection method	Vendor
F9-cap-F	ACCCGACAAGGAACAATGGT	Detection of FCV genome	SYBR Green	Fasmac (Atsugi, Japan)
F9-cap-R	GAGCAAAGGGCCTAAGGATTGT	Detection of FCV genome	SYBR Green	Fasmac
fβ-actin-F	TGCGTGACATCAAGGAGAAG	feline β-actin for normalization	SYBR Green	Fasmac
fβ-actin-R	AGGAAGGAAGGCTGGAAGAG	feline β-actin for normalization	SYBR Green	Fasmac
MNV-VP1-F	GCTACATTGCCCTGTCCTATTC	Detection of MNV genome	Probe	Integrated DNA Technologies (IDT, Tokyo, Japan)
MNV-VP1-probe	56-FAM/CACCTTCCC/ Zen/GACTGATGGCTTCTT/3lABkFQ	Detection of MNV genome	Probe	IDT
MNV-VP1-R	CCCACAGAGGCCAATTGATAA	Detection of MNV genome	Probe	IDT
mβ-actin-F	GATTACTGCTCTGGCTCCTAG-	mouse β-actin for normalization	Probe	IDT
mβ-actin-probe	56-FAM/CTGGCCTCA/ Zen/CTGTCCACCTTCC/3lABkFQ	mouse β-actin for normalization	Probe	IDT
mβ-actin-R	GACTCATCGTACTCCTGCTTG-	mouse β-actin for normalization	Probe	IDT
